# Acarbose, 17-α-estradiol, and nordihydroguaiaretic acid extend mouse lifespan preferentially in males

**DOI:** 10.1111/acel.12170

**Published:** 2013-11-19

**Authors:** David E Harrison, Randy Strong, David B Allison, Bruce N Ames, Clinton M Astle, Hani Atamna, Elizabeth Fernandez, Kevin Flurkey, Martin A Javors, Nancy L Nadon, James F Nelson, Scott Pletcher, James W Simpkins, Daniel Smith, J Erby Wilkinson, Richard A Miller

**Affiliations:** 1 The Jackson LaboratoryBar Harbor, ME, 04609, USA; 2 Barshop Institute for Longevity and Aging Studies, The University of Texas Health Science Center at San AntonioSan Antonio, TX, 78245, USA; 3 Geriatric Research, Education and Clinical Center, South Texas Veterans Health Care SystemSan Antonio, TX, 78229, USA; 4 Research Service, South Texas Veterans Health Care SystemSan Antonio, TX, 78229, USA; 5 Department of Pharmacology, The University of Texas Health Science Center at San AntonioSan Antonio, TX, 78229, USA; 6 Department of Biostatistics, University of Alabama at BirminghamBirmingham, AL, 35294, USA; 7 Children’s Hospital Oakland Research Institute5700 Martin Luther King Jr. Way, Oakland, CA, 94609-1673, USA; 8 Department of Psychiatry, The University of Texas Health Science Center at San AntonioSan Antonio, TX, 78229, USA; 9 Division of Aging Biology, National Institute on AgingBethesda, MD, 20892, USA; 10 Department of Physiology, The University of Texas Health Science Center at San AntonioSan Antonio, TX, 78229, USA; 11 Department of Molecular and Integrative Physiology, and Geriatrics Center, University of MichiganAnn Arbor, MI, 48109, USA; 12 Department of Pharmacology & Neuroscience, University of North Texas Health Science CenterFort Worth, TX, 76107, USA; 13 Department of Nutrition Sciences, University of Alabama at BirminghamBirmingham, AL, 35294, USA; 14 Unit for Laboratory Animal Medicine, University of Michigan School of MedicineAnn Arbor, MI, 48109, USA; 15 Department of Pathology and Geriatrics Center, University of MichiganAnn Arbor, MI, 48109, USA

**Keywords:** acarbose, estradiol, heterogeneous mice, lifespan, methylene blue, NDGA

## Abstract

Four agents — acarbose (ACA), 17-α-estradiol (EST), nordihydroguaiaretic acid (NDGA), and methylene blue (MB) — were evaluated for lifespan effects in genetically heterogeneous mice tested at three sites. Acarbose increased male median lifespan by 22% (*P* < 0.0001), but increased female median lifespan by only 5% (*P* = 0.01). This sexual dimorphism in ACA lifespan effect could not be explained by differences in effects on weight. Maximum lifespan (90th percentile) increased 11% (*P* < 0.001) in males and 9% (*P* = 0.001) in females. EST increased male median lifespan by 12% (*P* = 0.002), but did not lead to a significant effect on maximum lifespan. The benefits of EST were much stronger at one test site than at the other two and were not explained by effects on body weight. EST did not alter female lifespan. NDGA increased male median lifespan by 8–10% at three different doses, with *P*-values ranging from 0.04 to 0.005. Females did not show a lifespan benefit from NDGA, even at a dose that produced blood levels similar to those in males, which did show a strong lifespan benefit. MB did not alter median lifespan of males or females, but did produce a small, statistically significant (6%, *P* = 0.004) increase in female maximum lifespan. These results provide new pharmacological models for exploring processes that regulate the timing of aging and late-life diseases, and in particular for testing hypotheses about sexual dimorphism in aging and health.

## Introduction

Interventions that improve mammalian lifespan may provide new insights into the physiological factors that modulate aging rate and may eventually lead to treatments useful in the clinic to improve human health with age. Interventions that improve lifespan also are important for basic research, because different models of delayed aging can be contrasted to distinguish what biological effects are essential in delaying aging.

The National Institute on Aging Interventions Testing Program (ITP) has previously reported significant increases in lifespan caused by aspirin and nordihydroguaiaretic acid (NDGA) in male mice (Strong *et al*., 2008) and by rapamycin in both male and female mice (Harrison *et al*., 2009; Miller *et al*., 2011; Wilkinson *et al*., 2012). The design of the ITP, presented at this URL: (http://www.nia.nih.gov/research/dab/interventions-testing-program-itp), uses genetically heterogeneous (UM-HET3) mice, the offspring of the CByB6F1 x C3D2F1 cross. This cross produces a broad set of genetically diverse individuals, thus minimizing the possibility that characteristics of a single inbred or F1 hybrid genotype might be confused with those of the species. The ITP includes parallel replication of protocols at three sites, the University of Texas Health Science Center at San Antonio (UT), University of Michigan (UM), and The Jackson Laboratory (TJL), using standard operating protocols that reproduce key elements of the environmental conditions at each site. Sufficient numbers of mice are used in each yearly cohort to give more than 80% power to detect an increase or decrease of 10% in mean lifespan, with respect to controls of the same sex, even if only two of the three sites can contribute data to the pooled analysis.

The interventions for this study were chosen for the following reasons:

- Acarbose (ACA) is a plausible calorie restriction mimetic. Archer (2003) suggests that the postmeal spike in glucose may contribute to aging. This spike is reduced by ACA (Balfour & McTavish, 1993). Acarbose has been used clinically to prevent postprandial hyperglycemia for many years, and there are several reports showing its ability to limit or prevent postprandial hyperglycemia in mice as well (Frantz *et al*., 2005; Kim *et al*., 2011; Miyamura *et al*., 2010). The glucose spike during a meal is blunted because acarbose inhibits α-glucosidases in the intestine, thereby slowing the digestion of starches and disaccharides to glucose. Acarbose does not sequester glucose, nor block its uptake—it blocks its release from complex polysaccharides. Like diet restriction (DR), chronic ACA treatment reduces body weight and body fat, and also improves glucose dysregulation associated with aging (Yamamoto & Otsuki, 2006). DR greatly increases lifespan of both male and female UM-HET3 mice, the cross used in the current study (Flurkey *et al*., 2010). Unlike DR, food intake is often increased, not reduced, during long-term ACA treatment (Yamamoto & Otsuki, 2006).
- 17-α-estradiol (EST) is considered to be a nonfeminizing estrogen with reduced affinity for estrogen receptors. This estrogen is neuroprotective against ischemia in animal models, as well as *in vitro* (Perez *et al*., 2005). It also protects against neurodegenerative effects in cell and animal models of Parkinson’s disease (Dykens *et al*., 2005) and cerebrovascular disease (Liu *et al*., 2005). In general, nonfeminizing estrogens should not cause the kinds of deleterious effects seen in chronic estrogen therapy. Also, 17-α-estradiol should mimic the effects of 17-β-estradiol, particularly on neural tissues that are independent of the classical estrogen receptors. Studies in other laboratories, in mice and in neuroendocrine cell lines, have suggested that some of the effects of 17-β-estradiol, particularly with respect to its CNS effects, may be due to actions independent of the classical estrogen receptor (Huggins *et al*., 1954; Korenman, 1969; Kneifel *et al*., 1982; Green *et al*., 1997). We hypothesized that 17-α-estradiol might mimic these receptor-independent effects and lead to health benefits in male mice.
- Methylene blue (MB) greatly increases *in vitro* fibroblast lifespan and increases activity of mitochondria complex IV (Atamna *et al*., 2008). It also protects against seizures and oxidative damage in rat brain (Furian *et al*., 2007), as well as protecting against radiation, a standard cause of oxidative damage (Chung & Nam, 1975).
- Nordihydroguaiaretic acid (NDGA; also called masoprocol) has both antioxidant and anti-inflammatory properties (Zhang *et al*., 2013). In a previous study, the ITP researchers showed that NDGA increases median lifespan in males but not females. This sexual difference was tentatively attributed to differences in NDGA pharmacodynamics, because NDGA levels in serum were much higher in males than in females (Strong *et al*., 2008). Thus, in this current study, we evaluated a range of concentrations in males and tested whether a higher dose of NDGA would increase blood levels and improve lifespan in females.

## Results

### Effects of treatments on lifespan and markers of aging

#### Acarbose

**Figure 1:**
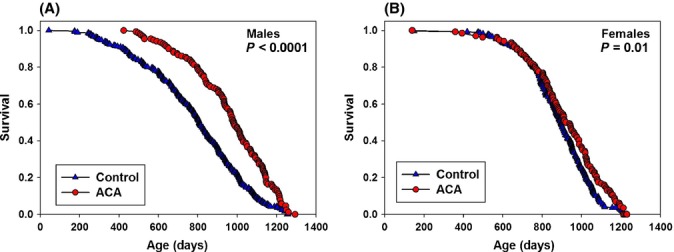
Survival curves comparing controls and mice fed Acarbose (ACA) for males (A) and females (B) pooled across the three sites. On this and other figures here, significance of differences between control and test mice are given as ‘*P’* values on each set of curves.

**Figure 2:**
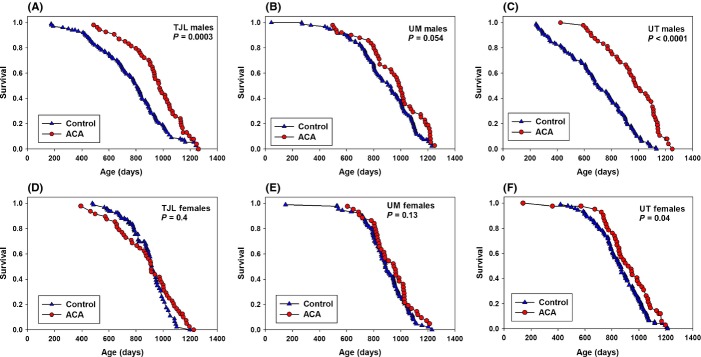
Survival curves comparing controls and mice fed Acarbose (ACA) for males (A,B,C) and females (D,E,F) from each of the three sites: TJL (A,D), UM (B,E), and UT (C,F).

Mice were fed ACA at 1000 mg kg^−1^ diet (1000 ppm) from 4 months of age. Male median lifespan was increased by 22% (*P* < 0.0001) and female median lifespan by only 5% (*P* = 0.01) (Fig. 1), using data pooled across sites. Figure 2 shows site-specific survival curves for both sexes. In male mice fed ACA (Fig. 2A–C), median lifespan was increased as follows: TJL (21%, 807–974 days, *P* = 0.0003); UM (8%, 925–999 days, *P* = 0.054); and UT (39%, 704–981 days, *P* < 0.0001). In sharp contrast, survival was affected far less in females fed ACA (Fig. 2D–F), with no significant effect at TJL (decreased from 918 to 911 days) or UM (increased from 887 to 949 days) and with a barely significant increase at UT (7%, 864–942 days, *P* = 0.04). Analyses of pooled and site-specific data are summarized in Table 1.

**Table 1:** Summary of survival curve information for ACA-, EST-, and MB-treated mice in Cohort 2009, in which all mice have died

	Median lifespan				Age at which 90% have died		
	Control mice (days)	Treated mice (days)	Difference (%)	Log-rank, *P* value	Control mice (days)	Treated mice (days)	Wang-Allison, *P* value
ACA: Males							
Pooled	807	984	22	<0.0001	1094	1215	<0.001
TJL	807	974	21	0.0003	1061	1212	0.09
UM	925	999	8	0.054	1051	1219	0.04
UT	704	981	39	<0.0001	1013	1204	<0.001
ACA: Females							
Pooled	896	939	5	0.0101	1072	1167	0.001
TJL	918	911	−1	0.4	1084	1148	0.01
UM	887	949	7	0.13	1089	1152	0.14
UT	864	942	9	0.04	1062	1167	0.11
EST: Males							
Pooled	807	900	12	0.002	1094	1148	0.13
TJL	807	828	3	0.6	1061	1117	0.8
UM	925	949	3	0.18	1151	1187	0.4
UT	704	900	28	0.0008	1013	1110	0.2
EST: Females							
Pooled	896	893	0	0.8	1072	1068	0.9
TJL	918	867	−6	0.9	1084	1107	0.5
UM	887	910	3	0.3	1089	1006	0.2
UT	864	903	5	0.6	1062	1079	0.4
MB: Males							
Pooled	807	790	−2	0.27	1094	1037	0.6
TJL	807	701	−13	0.07	1061	1009	0.2
UM	925	905	−2	0.96	1151	1157	0.8
UT	704	699	1	0.9	1013	980	0.9
MB: Females							
Pooled	896	902	1	0.17	1072	1138	0.004
TJL	918	862	−6	0.9	1084	1121	0.2
UM	887	993	12	0.048	1089	1159	0.01
UT	864	880	2	0.8	1062	1075	0.4

Pooled data represent all mice without adjustment for numbers at each site. The individual site values (TJL, UM, UT) are given below the pooled values. Medians of controls are repeated for comparison with medians of treated mice. Log-rank *P*-values consider all the data, while the Wang–Allison test evaluates the proportion of live mice in control and treatment groups at the age in days at which 90% of the ice have died.

Unlike survival curves and median lifespan, maximum lifespan (measured as the 90th percentile) in pooled data was increased to a similar degree in males and females; ACA caused an increase of 11% (*P* < 0.001) for males and 9% (*P* = 0.001) for females (Table 1). Giving site-specific data, in male mice fed ACA, maximum lifespan increased 14% at TJL, 6% at UM, and 19% at UT. Maximum lifespan in females fed ACA increased 6% at TJL, 6% at UM, and 10% at UT (Table 1). At TJL, there were many early deaths in females, but preferential survival for ACA in the oldest female mice. For UT females, results of the log-rank test were significant, but there was no significant effect on the late-life survival. For UM females, the trend was similar to that seen at UT, but the effect was less dramatic than at UT.

Interestingly, while survival of control UM-HET3 males was shorter at TJL and UT than at UM (*P* < 0.001; Fig. S1A), survival of ACA males increased most dramatically at TJL and UT, so that survival of ACA males did not differ among sites (*P* > 0.5; Fig. S1C). Survival of control UM-HET3 females did not differ among sites (*P* > 0.3; Fig. S1B) and also did not differ with ACA (*P* > 0.6; Fig. S1D).

**Figure 3:**
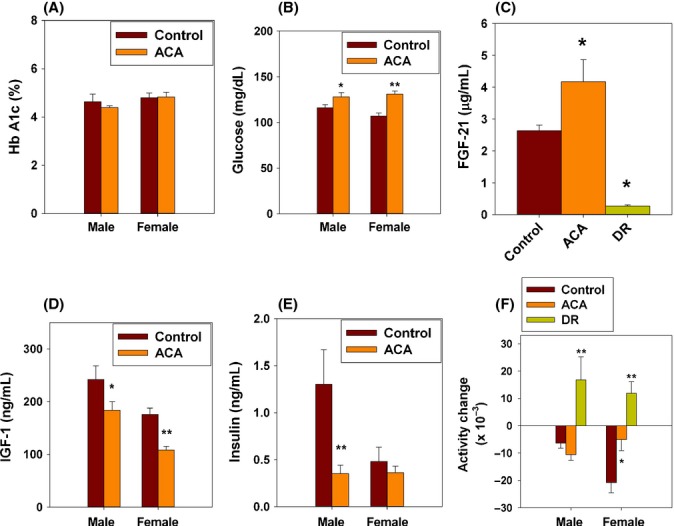
Data from an independent group of mice fed the standard Acarbose (ACA) diet starting at 4 months of age, tested at UM only. (A–E) Results from blood tests at 9 months of age: HbA1c, fasting glucose, FGF21, IGF1, and fasting insulin. (F) Activity, scored at 11 months as the number of beam interruptions in 50 h, adjusted for each mouse as a change score with reference to activity at 5 months for the same mouse. Diet restriction (C,F) was started at 4 months; mice were fed 60% of their normal consumption after a 2-week transitional period at 80%. Each bar shows mean and standard error of the mean. Numbers of mice: (A,B,D,E) *n* = 17–20 of each sex; (C) *n* = 10 per group, sexes pooled because there was no sex difference; (F) *n* = 17–18 males, 20 females.

In a separate group of mice, not involved in the lifespan study, ACA was administered from 4 months of age, and several physiological indices were evaluated over the next 8 months (Fig. 3). Hemoglobin A1c (Hb A1c) was used as an integrated measure of blood glucose levels over the previous 6 weeks and showed no effects of sex or of ACA (Fig. 3A). Fasting blood glucose levels were higher in ACA-fed males (*P* = 0.048) and females (*P* ≤ 0.0001) (Fig. 3B), consistent with the idea that ACA may delay the digestion of complex carbohydrates and absorption of sugars from the gastrointestinal tract. FGF21, a hormone produced by liver in response to fasting, extends lifespan when over-expressed in B6 mice (Zhang *et al*., 2012). Plasma FGF21 levels, measured in the morning after overnight fasting in 9-month-old mice, were elevated by ACA, but were greatly reduced by DR (Fig. 3C), showing a major difference in effects of ACA compared with DR. Figure 3D shows that IGF1 levels in plasma were significantly reduced by ACA in UM-HET3 males (*P* = 0.02) and females (*P* = 0.0001). Figure 3E shows that ACA reduced fasting insulin significantly in males (*P* < 0.001) but not in females (*P* = 0.6). The age-associated decline in voluntary activity from 5 to 11 months was reduced in females (*P* = 0.007) by ACA feeding, but was not affected in males. DR mice showed an increase in activity in these young adult mice of both sexes (Fig. 3F), showing another difference between effects of ACA and DR.

**Figure 4:**
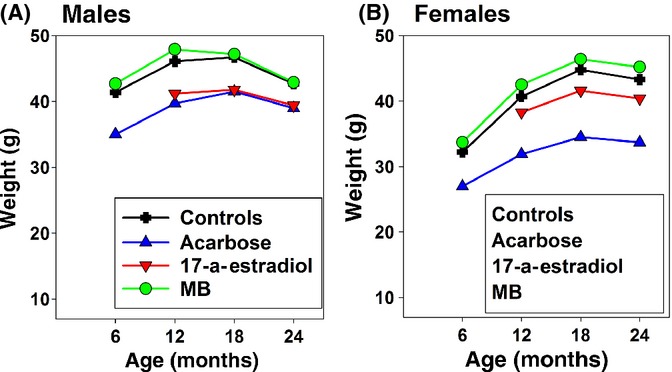
Weights are given for males (A) and females (B) at 6-month intervals for controls and mice fed Acarbose (ACA), EST, and MB. Data were pooled from the three sites.

Acarbose reduced body weights considerably more in females than in males: At 6, 12, 18, and 24 months of age, males were 15%, 14%, 11%, and 9% lighter than controls, while females were 15%, 22%, 23%, and 22% lighter, respectively (Fig. 4). Only pooled data are shown in the figure; data from each site were similar (Fig. S2). In all cases, loss of weight due to ACA was as high or higher in females than in males.

#### 17-α-estradiol (EST)

**Figure 5:**
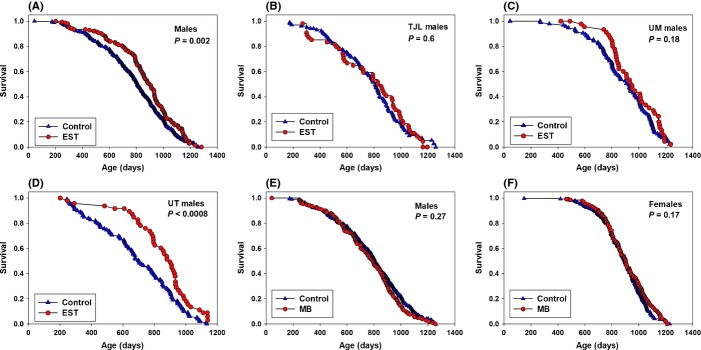
Survival curves comparing controls and mice fed EST for males pooled from the three sites (A) and for males at each site: TJL (B), UM (C), and UT (D). Survival curves for males and females fed MB pooled from the three sites are (E) and (F), respectively.

One group of mice was fed 4.8 mg EST/kg diet from 10 months of age. In males fed EST, median lifespan increased only 3% at TJL and at UM (not significant), but at UT, lifespan increased 28%, (*P* = 0.0008; Fig. 5B–D; Table 1). Pooled median lifespan increased 12% (*P* = 0.0012; Fig. 5A), clearly due to the very strong benefits on male lifespan from EST at UT (Fig. 5D). There was no significant effect on maximum lifespan in males. EST feeding had no significant effect on female survival either in the pooled data or at any individual site (Table 1; Fig. S3). At 12, 18, and 24 months of age, males fed EST were 11%, 10%, and 8% lighter than controls (Fig. 4A), while females fed EST were 6%, 7%, and 7% lighter than controls (Fig. 4B).

Although EST is considered to be nonfeminizing, there is evidence that it has similar affinity for estrogen receptors a and b (Toran-Allerand *et al*., 2005) and can have uterotrophic effects (Clark *et al*., 1982). We therefore tested for feminizing activity of the EST diet in a separate population of young ovariectomized UM-HET3 mice at UT. EST fed to ovariectomized mice for 6 weeks did not increase uterine weight or cause cornification of the vaginal epithelium, indicating that at the dose used in this study, EST had no detectable bioactivity in the female reproductive tract.

#### Nordihydroguaiaretic acid (NDGA)

**Figure 6:**
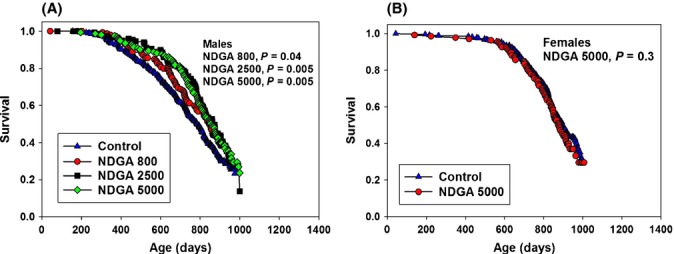
Survival curves comparing male controls and male mice fed NDGA at three different doses (A). Data for female controls and female mice fed the high dose of NDGA (B). Data were pooled from the three sites.

Starting at 6 months of age, male UM-HET3 mice were fed NDGA at three different doses, 800, 2500, or 5000 mg kg^−1^ diet (referred to as low, middle, or high-dose NDGA). Median survival of males was increased significantly at each of the three doses, as evaluated using the log-rank test. Estimates of the 90th percentile survival and statistical tests of maximum lifespan are not yet available from this cohort of mice. The middle dose led to increased male lifespan in a prior study (Strong *et al*., 2008) and is replicated in our current independent cohort. Figure 6A shows male interim survival curves pooled from all three sites. At the time of writing, about 30% of the cohort is still alive, and the youngest mice are approximately 850 days old; thus, the exact shapes of the survival curves shown in Fig. 6 are not defined above 850 days. In data pooled across sites, median lifespan for male controls was 787 days and was increased to 851 (*P* = 0.04), 868 (*P* = 0.0053), and 860 (*P* = 0.0048) days in groups fed low, middle and high doses of NDGA. NDGA was fed to females only at the high dose, 5000 mg kg^−1^, and female survival curves, about 70% complete, were not affected (Fig. 6B), with control and high-dose NDGA median lifespan 897 and 880 days, respectively. This is consistent with our previous study, in which female lifespan was not affected by NDGA at 2500 mg kg^−1^, the middle dose in males in the current study. Interim survivals at the three sites are similar (Fig. S4), except that the high dose may reduce longevity slightly at UM while the high dose is the best of the three at increasing survival at TJL and UT. As in the previous cohort, survival of male controls at TJL and UT is much shorter than at UM in the current cohort of mice.

Starting at 6 months of age, a cohort of male and female UM-HET3 mice at each site were fed diets containing different concentrations of NDGA. At 23–25 months of age, the mice were bled and the samples sent to the UT site for measurement of NDGA blood levels. Whole blood NDGA was proportional to the dose fed male mice: 140, 55, and 25 ng mL^−1^ plasma at the high, middle, and low dose, respectively. In female mice fed the high dose, the plasma concentration of NDGA was 60 ng mL^−1^, similar to that in males fed the middle dose (Fig. S5).

#### Methylene blue (MB)

Starting at 4 months of age, UM-HET3 mice were fed 27 mg MB per kg diet. Survival curves for pooled male or female data were not changed significantly by MB (Fig. 5E,F). Male survival was not affected at any of the three sites, although there was a suggestion (*P* = 0.07) of a negative effect at TJL, with a 13% decline in median survival.

Female median survival was not affected at TJL or UT; median lifespan increased 12% (*P* = 0.048) at UM (Table 1, Fig. S7). While statistically significant, we do not consider this of biological significance, especially because the effect of MB in female data pooled from the three sites is only a nonsignificant 1% increase in lifespan (Fig. 5F). However, maximum lifespan was increased at both TJL and UM, so a 6% increase in females using pooled data (90th percentile age of 1072 in controls and 1138 days in MB females) was significant (*P* = 0.004; Table 1).

### Effects of treatments on lesions and inferred causes of death

Table S1 summarizes numbers of different lesions that are inferred causes of death. Of the 128 cases for which the pathologist could infer a single specific cause of death, there were no significant differences between control and ACA-treated mice or between control and EST-treated mice in the proportion of mice dying with any cause of death, probably because so many different causes are represented. In addition to specifying a likely cause of death, the pathologist also evaluated the severity of age-related ‘incidental’ lesions, that is, lesions that did not lead to death. In this analysis, the incidence of liver degeneration was reduced by ACA from 42% to 3% (*P* = 0.001) in males, but ACA did not have a similar effect in females, with 17% liver degeneration in ACA-treated and 13% in controls. It is possible that improved liver function in ACA-treated males may have contributed, in unknown ways, to their improved survival. ACA increased the incidence of cold thyroid follicles (from 78% to 92%, *P* = 0.05) with no effect of sex. This significance level is borderline in a series of multiple comparisons. Hepatic lipidosis (‘macro lipidosis’) is diminished significantly by ACA (from 27% to 4%, *P* = 0.002) in the pooled data. In males, ACA reduces this finding, *P* = 0.02, while the incidence is so low in control females that the ACA effect is not significant. EST has a similar effect, with a reduction from 27% to 6% (*P* = 0.006) in pooled data, and *P* = 0.01 in males alone, but there is no significant effect in females. Because this type of lesion is more common in males than in females, its reduction also may help explain the unbalanced benefits of ACA and EST on male survival.

### Overall patterns did not change when early deaths were removed

Due to significant early deaths in males at UT and TJL, probably related to mouse urinary syndrome (MUS) (Tuffery, 1966; Everitt *et al*., 1988), male survival statistics were recalculated after eliminating all deaths at ages earlier than 600 days, to estimate survival effects in those mice that reach at least 600 days, ~ 20 months of life (Table S2). Overall, the findings were similar to those in males with all deaths considered: significant positive effects of ACA and EST, but not MB. Exclusion of males dying before 600 days reduces the percentage effect of ACA (from 22% to 13%) and EST (from 12% to 5%), but the log-rank tests are significant even with lower values of N. In these left-censored data sets, ACA had a significant benefit at TJL and UT, with a similar nearly significant pattern (*P* = 0.055) at UM. In the truncated data sets taken at individual sites, EST had a significant benefit only at UT, similar to the findings with the complete data sets shown earlier.

## Discussion

Interventions that increase lifespan may be doing so by retarding aging and, thus, may provide powerful tools for analyzing the biology of aging and late-life diseases. Data showing that a drug can increase lifespan are the first step in developing the case that the drug can delay or decelerate aging. Repeatable evidence that mouse lifespan can be extended by drugs has developed only recently (Strong *et al*., 2008; Harrison *et al*., 2009; Miller *et al*., 2011), and the question of whether these lifespan effects do indeed reflect slower aging is under active investigation (Chen *et al*., 2009; Wilkinson *et al*., 2012). Here, we report strong evidence for lifespan extension by ACA, evidence for a possible effect of EST, and strong confirmatory evidence for benefits from NDGA. Surprisingly, each of these three agents extends lifespan either in males only or (for ACA) much more strongly in males than in females. These data thus provide new systems for exploring questions about sex-specific processes that regulate lifespan and late-life illnesses.

Lifespan studies provide a valuable screening tool to identify agents (e.g., drugs or diets) that might slow aging and thereby delay the onset of the wide range of age-related diseases, including those that lead to death. It is, however, a realistic possibility that an agent might extend lifespan by an effect on a specific kind of lethal illness, such as the neoplastic diseases that are the usual cause of death in laboratory mouse stocks. Further work on a range of age-sensitive changes, in many cells and tissues, are needed to determine whether NDGA, ACA, or EST are extending longevity by retarding basic mechanisms of aging, by postponing death from multiple forms of neoplasia, or by a combination of these. The ITP protocol uses genetically heterogeneous mice to minimize the risk of confusion due to unique characteristics of single inbred or isogenic genotypes. Further studies of ACA, EST, and NDGA in a wide variety of mouse stocks will be of high interest, to identify pathways and genetic contributors to the lifespan extension effects.

One possible reason for the larger or exclusive effects of these compounds on males is the short lifespan of the male controls at two of the three sites (medians of 704, 807, and 924 days from male controls at UT, TJL, and UM, respectively, while female control medians are 864, 918, and 887, Table 1). As in all previous ITP cohorts (Strong *et al*., 2008; Harrison *et al*., 2009; Miller *et al*., 2011), unknown site-specific differences lead to male control mice living longer at UM than at UT and TJL, without producing corresponding discrepancies among female controls. We do not know why this occurs, and we do not know whether the factors that distinguish TJL from UM males are the same as those that distinguish UT from UM males. If we knew the basis for the site-specific effect on control lifespan, and why it affects only males, we would be able to test the idea that the stronger (proportional) response of males to interventions such as ACA at TJL and UT represented a correction of the postulated sex-and-site-specific factor(s). We cannot rule this idea out, but we also cannot test it without a plausible hypothesis as to the nature of the factor(s) involved. The data at hand suggest that the situation may be complex and not attributable to a single factor.

Acarbose (ACA). We expected ACA to mimic many effects of diet restriction (DR). The diet used in these studies, Purina 5LG6 diet, has high starch and low sucrose. We expected that ACA, by slowing complex carbohydrate digestion, might limit absorption of sugars and simple carbohydrates, thus leading to lower overall caloric intake and producing effects similar to those of DR. DR at 2/3 normal food consumption started at 4–5 weeks of age increased survival of UM-HET3 mice, with mean lifespan increasing 40% (836–1169 days) in females and 32.5% (831–1101 days) in males; DR decreased body weight to about half in both sexes (Flurkey *et al*., 2010). Thus, we expected that ACA might produce some survival benefits, although not as much as produced by DR, because the weight reductions from ACA (Fig. 4) were much less than those seen in DR mice. In addition, as weight reductions (% body weight) due to ACA were greater in females than in males (Fig. 4), we hypothesized that lifespan effects would, similarly, be larger in females. The results were thus surprising: median lifespan was increased 22% in males and only 5% in females (Fig. 1).

The fact that female control lifespans do not differ at the three sites, but are still increased significantly by ACA, suggests that ACA may affect basic mechanisms of aging that affect survival in both sexes. In addition, it is noteworthy that the effects of ACA in male mice at UM, where male controls are longest-lived, approached or met conventional criteria for statistical significance (*P* = 0.054 for log-rank test; *P* = 0.04 for Wang/Allison test of 90th percentile survival) despite the limited statistical power at any single test site. The idea that ACA has effects on aging and survival independent of site-specific environmental factors is further supported by the fact that in both males and females, maximum lifespan (90th percentile) is increased significantly and similarly in the pooled data for both males (11%, *P* < 0.001) and females (9%, *P* = 0.001) (Table 1). Maximum lifespan often indicates effects on mechanisms of aging more reliably than mean or median lifespan, as the latter may be altered by early deaths unrelated to aging.

Acarbose reduced weight more in females than in males (Fig. 4). Thus, the lengthened survival for ACA-treated males vs. ACA-treated females cannot be explained by changes in body weight or seen simply as the effect of overall caloric restriction. It is not currently known whether slower weight gain is a direct effect of loss of caloric content absorbed or represents a modulation of CNS and gastrointestinal endocrine circuits, perhaps with modification of appetite and associated metabolic set points. The data in Fig. 3 provide an initial indication of key physiological parameters in young adult ACA-treated mice. The lower fasting glucose values, in combination with the unaltered HbA1c levels, are consistent with the idea that ACA may diminish the amplitude of postprandial spikes in plasma glucose levels, with lower peak levels but higher trough levels in both sexes. In both blood levels of FGF21 and activity, effects of ACA differed from effects of DR (Fig 3C,F), and thus benefits of ACA on lifespan may not be attributed simply to diminished caloric intake. The male-specific decline in fasting insulin level hints that this altered diurnal pattern of glucose spikes may produce higher insulin sensitivity in males, with less effect in females, which might in turn contribute to the sexual dimorphism in longevity effect. Furthermore, differences between ACA and CR may be due to carbohydrate vs. total diet restriction or to changes in the microbiome. Each of these ideas warrants more thorough evaluation at different ages and in different tissues involved in glucose homeostasis. ACA-treated mice also had a mild reduction in plasma IGF1 levels, of equivalent size in both sexes. Yuan *et al*. (2012) showed a strong association among mouse strains between levels of IGF1 at 6 months of age and lifespan, but this association disappeared when IGF1 was tested at 12 and 18 months of age. There is some evidence that IGF1 levels at 6 months may predict lifespan in F1 hybrids (Harper *et al*., 2006) as well as in UM-HET3 mice, where low levels at 15 months predicted increased lifespan (Harper *et al*., 2003).

17 α-estradiol (EST) was suggested as an anti-aging intervention on the grounds that it might mimic, in male mice, the beneficial effects produced by estrogen in control females and might avoid the serious adverse effects of chronic estrogen treatment mediated by activation of estrogen receptors. Thus, rather than using hormonally active estrogen, we used a nonfeminizing estrogen with reduced binding for estrogen receptors. This class of nonfeminizing estrogen has shown many health benefits (Dykens *et al*., 2005; Liu *et al*., 2005; Perez *et al*., 2005), and non-feminizing estrogens should not cause deleterious effects seen in chronic estrogen therapy (Wang *et al*., 2006). EST feeding was started at 10 months of age in mice, at the age when estrus cycling and reproduction begin to slow down in long-lived females (Nelson *et al*., 1982). Evidence for effects on aging was inconclusive. EST feeding had no effect on lifespan in females, as expected. Although the pooled data showed a significant longevity benefit in males, the magnitude of this effect was far stronger at UT than at the other two test sites (Table 1, Fig. 5). Interestingly, weights were affected by EST to a similar degree at TJL and UT (Fig. S2), although a large increase in survival only occurred at UT. Thus, the reduction in weight in males does not consistently explain the great effect of EST on male survival at UT. The test of the hypothesis that 17-α-estradiol might improve health in males thus gives equivocal results—a dramatic benefit in males at one site but no significant benefits at the other two sites. More work will be needed to see whether or not the EST result is a site-specific observation. If the health benefits males at UT are replicated, perhaps by other groups, on other stocks, or at higher doses of 17-α-estradiol, this should motivate follow-up studies to learn which cell type(s) and which estrogen-dependent/independent events are responsible for the benefit.

Nordihydroguaiaretic acid (NDGA) has been shown in a previous cohort to increase lifespan in male but not female UM-HET 3 mice (Strong *et al*., 2008). We tested it a second time because NDGA serum concentrations were, in the original study using 2500 ppm NDGA, much lower in females than in males, which might explain the absence of lifespan benefit in females. In the current study, we evaluated a higher dose of NDGA (5000 ppm) in an attempt to increase blood levels to see whether these would increase female lifespan. The blood level was indeed increased in females receiving 5000 ppm to a value similar to that in males receiving 2500 ppm NDGA (Fig. S5), but we saw no evidence for an increase in female lifespan with 70% of the survival curve complete (Fig. 6). Effects in males (Fig. 6) were similar to those reported in our earlier study (Strong *et al*., 2008), and the medium dose of NDGA used previously was the most consistent at increasing lifespan (Figs. 6 and S4). The new data confirm the original report in an independent cohort, show that the lack of effect in females is not due simply to altered pharmacodynamics, and provide helpful information on the dose–response relationship. Although the current NDGA survival experiment is not complete, it is apparent that NDGA, such as ACA and EST, has a significant benefit in males but not in females. In males, the low and middle doses did not affect body weight, but the high dose caused a small reduction (Fig. S6). The high dose reduced female body weight about 14%, 22%, and 23% at 12, 18, and 24 months, respectively, while male weight was only reduced 3–6% (Fig. S6B); thus, the lack of effect of NDGA on female lifespan cannot be explained by concentration in the plasma or by effects on body weight. It is possible that NDGA produces beneficial effects in both sexes but produces harmful effects in females that prevent lifespan extension. Further data, in both sexes, on age-dependent changes, including those related to overall health, will be needed to determine whether NDGA modulates multiple aspects of aging in either or both sexes. It is noteworthy that several of the agents found to extend lifespan in the ITP series, that is, aspirin, NDGA, and rapamycin, inhibit some forms of inflammatory response, consistent with suggestions that late-life inflammation is an important aging process (Jenny, 2012). A mutation that blocks production of the pro-inflammatory cytokine MIF (migration inhibition factor) also extends mouse lifespan (Harper *et al*., 2010).

Methylene blue (MB) extends proliferative lifespan of human embryonic fibroblasts (IMR90) and also increases the activity of mitochondrial complex IV as well as mitochondrial heme synthesis in these fibroblasts; it reverses accelerated senescence *in vitro* due to oxidative stress and induces antioxidant defense enzymes in HepG2 cells (Atamna *et al*., 2008). Despite this, and the protection against irradiation, brain damage, poisoning, and chemotherapy *in vitro,* MB did not lead to strong or consistent effects on lifespan in UM-HET 3 mice at the dose used, although it did lead to a statistically significant improvement in female maximum lifespan (Fig. 5F), as measured by the proportion of surviving mice at the 90th percentile age in the pooled data set. Specific effects on median lifespan at a single site suggest damage in males (TJL) or benefits in females (UM), but these effects disappear in pooled data (Table 1). There is a 6% increase in female maximum 90% lifespan that is significant (Table 1). However, because the effect is so small, and limited to one sex, the data fail to support the hypothesis that MB is an effective anti-aging intervention at the dose used.

We evaluated slope and intercept of the Gompertz distribution for males and females exposed to EST, ACA, and MB as previously described (Miller *et al*., 2011). For females, pooling across sites, there were no significant effects of any of these agents on the slope or intercept coefficients. For males exposed to ACA, slope is increased and intercept is decreased, although interpretation of these effects is complicated by the high sensitivity of both parameters to early deaths whose relationship to aging processes is obscure. Neither MB nor EST led to a significant alteration in either Gompertz parameter.

We believe our new data provide several important steps toward the understanding of aging mechanisms and the search for clinical strategies to retard chronic diseases. ACA represents the second agent, among those tested by the ITP, that significantly increases both male and female lifespan. Compared with rapamycin, ACA produces larger median effects and similar 90th percentile effects in males; effects in females are much less than produced by rapamycin (Harrison *et al*., 2009; Miller *et al*., 2011). The ACA result draws attention to the possibility that transient fluctuations in plasma glucose, thought to be blunted in ACA-treated mice, may play a role in aging and in cancer biology. Comparisons of ACA to various food restriction protocols are likely to be quite informative: both ACA and DR lead to longevity, some change in body mass (much more severe in DR mice), and alterations in glucose/insulin physiology, but the two protocols differ in fasting glucose, stimulation of spontaneous activity and perhaps other CNS responses, post-prandial glucose pulse height, and in the sexual specificity of the lifespan response. Our data show that drugs can lead to dramatically different effects on lifespan in the two sexes, with ACA, NDGA, and (perhaps) EST showing benefits in males, and rapamycin showing consistently stronger effects in females (Harrison *et al*., 2009; Miller *et al*., 2011, and unpublished results). The hints of benefits from MB suggest that MB might lead to lifespan and anti-aging benefits using other doses or alternate dosage schedules or both. More generally, the growing arsenal of drugs that extend lifespan, perhaps by modulation of aging, cancer, or both, will complement work performed using mutant stocks and dietary interventions to delineate the factors that control aging rate in mammals and link aging to multiple forms of illness.

## Experimental procedures

### Mouse production, maintenance, and estimation of lifespan

UM-HET3 mice were produced at each of the three test sites as previously described (Strong *et al*., 2008; Harrison *et al*., 2009; Miller *et al*., 2011), where environmental conditions are presented in detail. The dams of the test mice were CByB6F1/J, JAX stock #100009 (dams, BALB/cByJ; sires, C57BL/6J). The sires of the test mice were C3D2F1/J, JAX stock #100004 (dams, C3H/HeJ; sires, DBA/2J). In each site, breeding mice were fed LabDiet® 5008 mouse chow (PMI Nutritional International, Bentwood, MO, USA). As soon as mice were weaned, they were fed LabDiet® 5LG6 from the same source.

Details of the methods used for health monitoring were provided previously (Strong *et al*., 2008; Harrison *et al*., 2009; Miller *et al*., 2011). In brief, each of the three colonies was evaluated four times each year for infectious agents. All such surveillance tests were negative for pathogens at all three sites throughout the entire study period.

### Removal of mice from the longevity population

Mice were removed from the study because of fighting or accidental death (e.g., during chip implantation) or chip failure, or because they were used for another experimental purpose, such as testing immune responses. For survival analyses, all such mice were treated as alive at the date of their removal from the protocol and lost to follow-up thereafter. These mice were not included in calculations of median longevity. Overall, 3–5% of the mice were removed from the longevity populations reported here, with no significant site differences, except that about 20% of female controls were removed at TJL to be used for a separate investigation.

### Estimation of age at death (lifespan)

At UM and UT, mice were examined daily for signs of ill health from the time they were set up in the experiment. At JAX, once mice were marked as ill, they were examined daily for signs of ill health. Mice were euthanized for humane reasons if so severely moribund that they were considered, by an experienced technician, unlikely to survive for more than an additional 48 h. A mouse was considered severely moribund if it exhibited more than one of the following clinical signs: (i) severe lethargy, as indicated by reluctance to move when gently prodded with a forceps; (ii) inability to eat or to drink, for example due to a severe balance or gait disturbance; (iii) rapid weight loss over a period of 1 week or more; or (iv) a severely ulcerated or bleeding tumor. The age at which a moribund mouse was euthanized was taken as the best available estimate of its natural lifespan. Mice found dead were also noted at each daily inspection.

### Control and experimental diets

TestDiet®, Inc., a division of Purina Mills (Richmond, IN, USA), prepared batches of LabDiet® 5LG6 food containing each of the test substances, as well as control diet batches, at intervals of approximately 4 months, and shipped each batch of food at the same time to each of the three test sites. Acarbose was purchased from Spectrum Chemical Mfg. Corp., Gardena, CA. It was mixed at a concentration of 1000 mg of ACA per kilogram of diet (1000 ppm); mice were fed continuously from 4 months of age. 17 α-estradiol (EST) was purchased from Steraloids, Inc. (Newport, RI, USA) and mixed at a dose of 4.8 mg kg^−1^ of diet (4.8 ppm); mice were fed continuously from 10 months of age. Methylene blue (MB) was purchased as methylene blue hydrate (Cat # 66720) from Sigma-Aldrich (St Louis, MO) and mixed at 28 mg kg^−1^ of diet (28 ppm); mice were fed continuously from 4 months of age. Nordihydroguaiaretic acid (NDGA) was purchased from Cayman Chemicals (Ann Arbor, MI, USA) and was mixed at concentrations of 800, 2500, or 5000 mg kg^−1^ of diet (800, 2500, 5000 ppm); mice were fed continuously from 6 months of age. Lifespan data for NDGA are not complete because these studies were started a year later than the others reported. NDGA treatment was in Cohort 2010 mice, while ACA, EST, and MB treatments were included in Cohort 2009.

### Measurement of NDGA

NDGA was quantified in mouse blood using HPLC with electrochemical detection. Briefly, 100 μL of calibrators, controls, and unknown samples were mixed with 0.9 mL of a 50% ACN/25% IPA/25% EtOH/0.1% ascorbic acid solution. The samples were vortexed vigorously and then centrifuged at 3200 *g* for 20 min. Supernatants were transferred to glass test tubes and dried to residue under a gentle stream of nitrogen. The residues were redissolved in 100 μL of mobile phase and then filtered using a microfilterfuge tube. Then, 50 μL of the final samples was injected into the HPLC-EC system. The peak area of NDGA was compared against a linear regression of peak areas of calibrators at concentrations of 0, 10, 50, 100, 500, and 1000 ng mL^−1^ to quantify NDGA in the blood samples. NDGA concentration in blood was reported in ng mL^−1^.

The HPLC-electrochemical system consisted of an Alltima C18 column (4.6 × 150 mm, 3 micrometer) heated to 36 °C, ESA Coulochem II Detector with 5011 detector cell, Waters 717 autosampler, and a Waters 515 HPLC pump. The mobile phase was 50% MeOH, 49.5% Milli-Q water, 0.1 mm EDTA, and 0.5% phosphoric acid (pH 2.5). The flow rate of the mobile phase was 0.75 mL min^−1^, and the detector settings were E1: +450 mV, R1: 100 nA, E2: −350 mV, R2: 100 nA, Guard Cell: +300 mV.

### Statistical methods

Each mouse originally entered into the study was, at the time of analysis, considered to be in one of two categories: either dead (from natural causes) or censored. Mice were censored at the age when they were no longer subjected to the mortality risks typical of unmanipulated mice. In some cases, this was because the mouse was removed because of fighting; in other cases, mice died as the result of an accident (e.g., death when anesthetized for implantation of a radio-emitting chip). In still other cases, mice were considered censored on the day in which they received an experimental treatment (such as blood sampling or tests of immune response) to which the control mice were not exposed. Kaplan–Meier analysis and log-rank comparisons among groups considered censored mice to be lost from follow-up on the day at which they were removed from the longevity protocol. There were no mice remaining alive in the ACA, EST, and MB groups at the time of the analyses reported here. Only about 70% of the mice given NDGA and controls had died when these analyses were performed, so only median data are reported.

Unless stated otherwise, all significance tests about survival effects are based upon the two-tailed log-rank test at *P* < 0.05, stratified by test site, with censored mice included up until their date of removal from the longevity population. Other statistical tests are described in the text; all *P*-values are two-tailed and reported without adjustment for multiple comparisons. Statistical claims related to maximum lifespan are based on the procedure of Wang *et al*. (2004), which uses the Fisher exact test to compare the proportions of surviving mice, in Control and Test groups, at the age corresponding to the 90th percentile for survival in the joint distribution of the Control and Test groups together. For the pooled data sets, surviving mice were enumerated at the 90th percentile age for each site separately, and these counts were combined for the overall Fisher exact test.

## Supporting Information

**Fig. S1** ACA treatment: Site-specific survival curves for UM-HET3 controls (A,B) and ACA-fed male (C) and female (D) mice. **Fig. S2** ACA, EST and MB treatment: weights for UM-HET3 controls and males (A–C) and females (D–F) at TJL (A,D), UM (B,E), and UT (C,F). **Fig. S3** EST treatment: Survival for UM-HET3 control females pooled across sites (A) and EST-treated females at TJL (B), UM (C), and UT (D). **Fig. S4** NDGA treatment: Interim survival for UM-HET3 controls and NDGA-treated males at TJL (A), UM (B), and UT (C), and UM-HET3 controls and NDGA-treated females at TJL (D), UM (E), and UT (F). **Fig. S5** NDGA levels in plasma of UM-HET3 controls and males treated with high (5000 ppm), middle (2500 ppm), or low dose (9800 ppm) of NDGA in the diet (A), and UM-HET3 controls and females treated with high dose of NDGA (B). **Fig. S6** NDGA treatment: Body weights for UM-HET3 controls and males treated with high, middle, and low dose of NDGA (A), and for UM-HET3 controls and females treated with high dose of NDGA (B). **Fig. S7** MB treatment: Survival curves for UM-HET3 controls and mice fed MB in the diet for males at TJL (A), UM (B), and UT (C), and for females at TJL (D), UM (E), and UT (F). **Table S1** ACA and EST treatment: Inferred causes of death in aging UM-HET3 controls and ACA- and EST-treated males and females **Table S2** Survival in UM-HET3 control and male mice that were treated with ACA, EST, and MB in the diet and that lived at least 600 days

## References

[b1] Archer VE (2003). Does dietary sugar and fat influence longevity?. Med. Hypotheses.

[b2] Atamna H, Nguyen A, Schultz C, Boyle K, Newberry J, Kato H, Ames BN (2008). Methylene blue delays cellular senescence and enhances key mitochondrial biochemical pathways. FASEB J.

[b3] Balfour JA, McTavish D (1993). Acarbose. An update of its pharmacology and therapeutic use in diabetes mellitus. Drugs.

[b4] Chen C, Liu Y, Liu Y, Zheng P (2009). mTOR regulation and therapeutic rejuvenation of aging hematopoietic stem cells. Sci. Signal.

[b5] Chung SO, Nam SY (1975). The radioprotective effect against gamma-irradiation of methylene blue in the rat with reference to serum enzymes and pancreatic protein fractions examined by isoelectric focusing. J. Radiat. Res. (Tokyo).

[b6] Clark JH, Williams M, Upchurch S, Eriksson H, Helton E, Markaverich BM (1982). Effects of estradiol-17α on nuclear occupancy of the estrogen receptor, stimulation of nuclear type II sites and uterine growth. J. Steroid Biochem.

[b7] Dykens JA, Moos WH, Howell N (2005). Development of 17alpha-estradiol as a neuroprotective therapeutic agent: rationale and results from a phase I clinical study. Ann. N. Y. Acad. Sci.

[b8] Everitt JI, Ross PW, Davis TW (1988). Urologic syndrome associated with wire caging in AKR mice. Lab. Anim. Sci.

[b9] Flurkey K, Astle CM, Harrison DE (2010). Life extension by diet restriction and N-Acetyl-L-Cysteine in genetically heterogeneous HET3 Mice. J. Gerontol. A Biol. Sci. Med. Sci.

[b10] Frantz S, Calvillo L, Tillmanns J, Elbing I, Dienesch C, Bischoff H, Ertl G, Bauersachs J (2005). Repetitive postprandial hyperglycemia increases cardiac ischemia/reperfusion injury: prevention by the alpha-glucosidase inhibitor acarbose. FASEB J.

[b11] Furian AF, Fighera MR, Oliveira MS, Ferreira AP, Fiorenza NG, de Carvalho Myskiw J, Petry JC, Coelho RC, Mello CF, Royes LF (2007). Methylene blue prevents methylmalonate-induced seizures and oxidative damage in rat striatum. Neurochem. Int.

[b12] Green PS, Bishop J, Simpkins JW (1997). 17α-estradiol exerts neuroprotective effects in SK-N-SH cells. J. Neurosci.

[b13] Harper JM, Wolf N, Galecki AT, Pinkosky SL, Miller RA (2003). Hormone levels and cataract scores as sex-specific, mid-life predictors of longevity in genetically heterogeneous mice. Mech. Ageing Dev.

[b14] Harper JM, Durkee SJ, Dysko RC, Austad SN, Miller RA (2006). Genetic modulation of hormone levels and life span in hybrids between laboratory and wild-derived mice. J. Gerontol. A Biol. Sci. Med. Sci.

[b15] Harper JM, Wilkinson JE, Miller RA (2010). Macrophage migration inhibitory factor knockout mice are long-lived and respond to caloric restriction. FASEB J.

[b16] Harrison DE, Strong R, Sharp ZD, Nelson JF, Astle CM, Flurkey K, Nadon NL, Wilkinson JE, Frenkel K, Carter CS, Pahor M, Javors MA, Fernandez E, Miller RA (2009). Rapamycin fed late in life extends lifespan in genetically heterogeneous mice. Nature.

[b17] Huggins C, Jensen EV, Cleveland AS (1954). Chemical structure of steroids in relation to promotion of growth of the vagina and uterus of the hypophysectomized rat. J. Exp. Med.

[b18] Jenny NS (2012). Inflammation in aging: cause, effect or both?. Discov. Med.

[b19] Kim JH, Kang MJ, Choi HN, Jeong SM, Lee YM, Kim JI (2011). Quercetin attenuates fasting and postprandial hyperglycemia in animal models of diabetes mellitus. Nutr. Res. Pract.

[b20] Kneifel MA, Leytus SP, Fletcher E, Weber T, Mangel WF, Katzenellenbogen BS (1982). Uterine plasminogen activator activity: modulation by steroid hormones. Endocrinology.

[b21] Korenman SG (1969). Comparative binding affinity of estrogens and its relation to estrogenic potency. Steroids.

[b22] Liu R, Wen Y, Perez E, Wang X, Day AL, Simpkins JW, Yang S-H (2005). 17 β-Estradiol attenuates blood-brain barrier disruption through by cerebral ischemia-reperfusion injury in female rats, brain. Research.

[b23] Miller RA, Harrison DE, Astle CM, Baur JA, Boyd AR, de Cabo R, Fernandez E, Flurkey K, Javors MA, Nelson JF, Orihuela CJ, Pletcher S, Sharp ZD, Sinclair D, Starnes JW, Wilkinson JE, Nadon NL, Strong R (2011). Rapamycin, but not resveratrol or simvastatin, extends life span of genetically heterogeneous mice. J. Gerontol. A Biol. Sci. Med. Sci.

[b24] Miyamura M, Schnell O, Yamashita C, Yoshioka T, Matsumoto C, Mori T, Ukimura A, Kitaura Y, Matsumura Y, Ishizaka N, Hayashi T (2010). Effects of acarbose on the acceleration of postprandial hyperglycemia-induced pathological changes induced by intermittent hypoxia in lean mice. J. Pharmacol. Sci.

[b25] Nelson JF, Randall PK, Sims C, Finch CE (1982). A longitudinal study of estrous cyclicity in aging C57BL/6J mice: I. Cycle frequency, length and vaginal cytology. Biol. Reprod.

[b26] Perez E, Liu R, Yang SH, Cai ZY, Covey DF, Simpkins JW (2005). Neuroprotective effects of an estratriene analog are estrogen receptor independent in vitro and in vivo. Brain Res.

[b27] Strong R, Miller RA, Astle CM, Floyd RA, Flurkey K, Hensley KL, Javors MA, Leeuwenburgh C, Nelson JF, Ongini E, Nadon NL, Warner HR, Harrison DE (2008). Nordihydroguaiaretic acid and aspirin increase lifespan of genetically heterogeneous male mice. Aging Cell.

[b28] Toran-Allerand DC, Tinnikov AA, Singh RJ, Nethrapalli IS (2005). 17? -Estradiol: a brain-active estrogen. Endocrinology.

[b29] Tuffery AA (1966). Urogenital lesions in laboratory mice. J. Pathol. Bacteriol.

[b30] Wang C, Li Q, Redden DT, Weindruch R, Allison DB (2004). Statistical methods for testing effects on “maximum lifespan”. Mech. Ageing Dev.

[b31] Wang X, Dykens JA, Perz E, Liu R, Yang S, Covey DF, Simpkins JW (2006). Neuroprotective effects of 17β-estradiol and non-feminizing estrogens against H2O2 toxicity in human neuroblastoma SK-N-SH cells. Mol. Pharmacol.

[b32] Wilkinson JE, Burmeister L, Brooks SV, Carames B, Friedline S, Harrison DE, Lotz M, Nadon N, Strong R, Wood LK, Woodward MA, Miller RA (2012). Rapamycin slows aging in mice. Aging Cell.

[b33] Yamamoto M, Otsuki M (2006). Effect of inhibition of alpha-glucosidase on age-related glucose intolerance and pancreatic atrophy in rats. Metabolism.

[b34] Yuan R, Meng Q, Nautiyal J, Flurkey K, Tsaih S-W, Krier R, Parker MG, Harrison DE, Paigen B (2012). Genetic co-regulation of age of female sexual maturation and lifespan through circulating IGF1 among inbred mouse strains. Proc. Natl Acad. Sci. USA.

[b35] Zhang Y, Xie Y, Berglund ED, Coate KC, He TT, Katafuchi T, Xiao G, Potthoff MJ, Wei W, Wan Y, Yu RT, Evans RM, Kliewer SA, Mangelsdorf DJ (2012). The starvation hormone, fibroblast growth factor-21, extends lifespan in mice. eLIFE.

[b36] Zhang H, Shen WJ, Cortez Y, Kraemer FB, Azhar S (2013). Nordihydroguaiaretic acid improves metabolic dysregulation and aberrant hepatic lipid metabolism in mice by both PPARα-dependent and -independent pathways. Am. J. Physiol. Gastrointest. Liver Physiol.

