# Supplementary material for: Acarbose, 17-α-estradiol, and nordihydroguaiaretic acid extend mouse lifespan preferentially in males
Source: Aging Cell. 2013 Nov 19;13(2):273–82. doi: 10.1111/acel.12170 (PMC3954939; doi:10.1111/acel.12170)
Supplement: Supplementary file 1 — Fig. S1 ACA treatment: Site-specific survival curves for UM-HET3 controls (A,B) and ACA-fed male (C) and female (D) mice. Fig. S2 ACA, EST and MB treatment: weights for UM-HET3 controls and males (A–C) and females (D–F) at TJL (A,D), UM (B,E), and UT (C,F). Fig. S3 EST treatment: Survival for UM-HET3 control females pooled across sites (A) and EST-treated females at TJL (B), UM (C), and UT (D). Fig. S4 NDGA treatment: Interim survival for UM-HET3 controls and NDGA-treated males at TJL (A), UM (B), and UT (C), and UM-HET3 controls and NDGA-treated females at TJL (D), UM (E), and UT (F). Fig. S5 NDGA levels in plasma of UM-HET3 controls and males treated with high (5000 ppm), middle (2500 ppm), or low dose (9800 ppm) of NDGA in the diet (A), and UM-HET3 controls and females treated with high dose of NDGA (B). Fig. S6 NDGA treatment: Body weights for UM-HET3 controls and males treated with high, middle, and low dose of NDGA (A), and for UM-HET3 controls and females treated with high dose of NDGA (B). Fig. S7 MB treatment: Survival curves for UM-HET3 controls and mice fed MB in the diet for males at TJL (A), UM (B), and UT (C), and for females at TJL (D), UM (E), and UT (F). Table S1 ACA and EST treatment: Inferred causes of death in aging UM-HET3 controls and ACA- and EST-treated males and females Table S2 Survival in UM-HET3 control and male mice that were treated with ACA, EST, and MB in the diet and that lived at least 600 days [file acel0013-0273-sd1.docx]

**Fig S1** ACA treatment: Site-specific survival curves for UM-HET3 controls (A,B) and ACA-fed male (C) and female (D) mice.

| **(A)** | **(B)** |
| --- | --- |
| 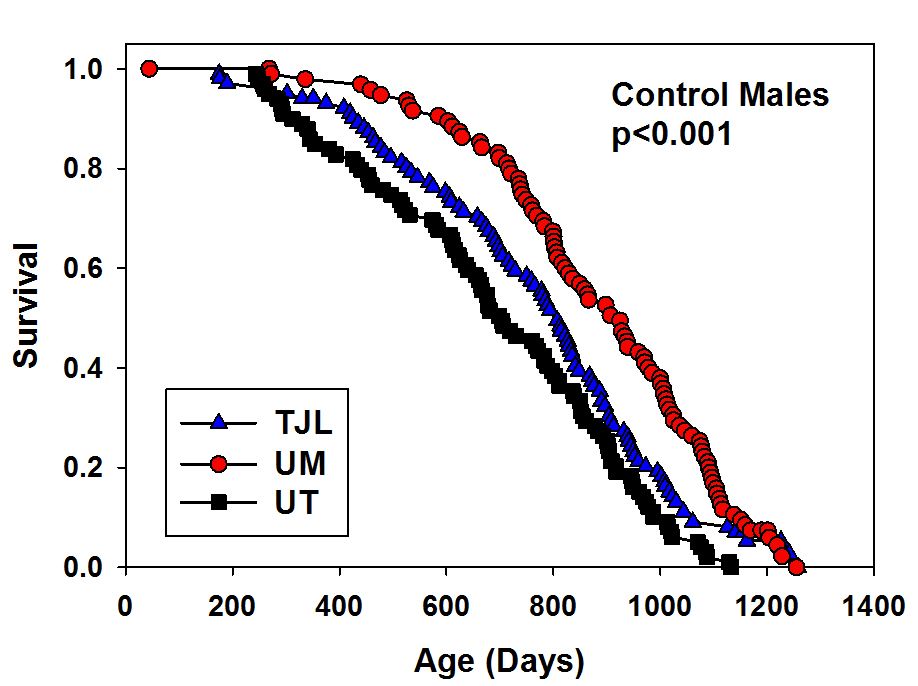 | 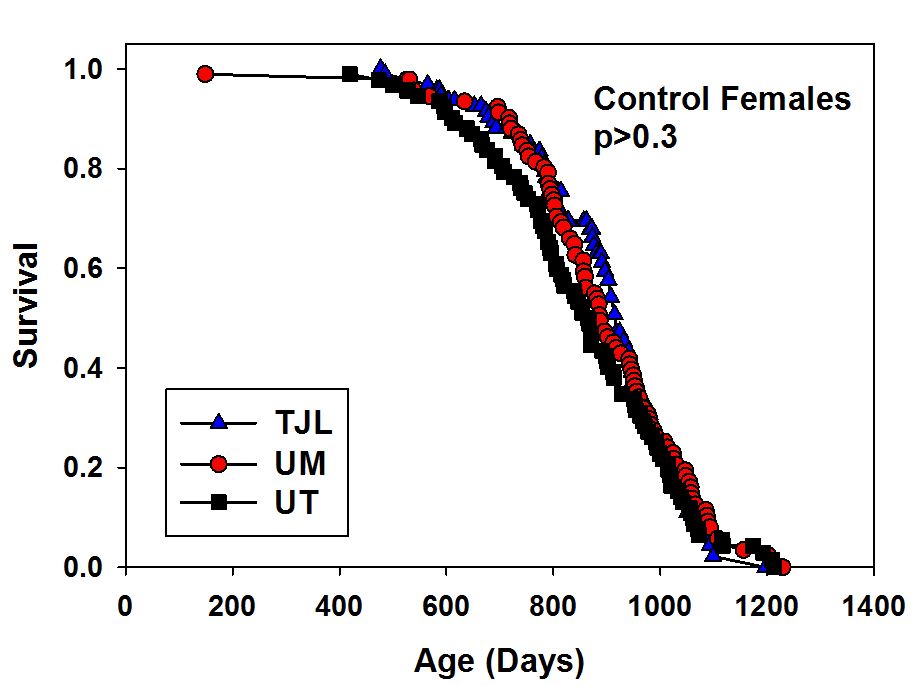 |
| **(C)** | **(D)** |
| 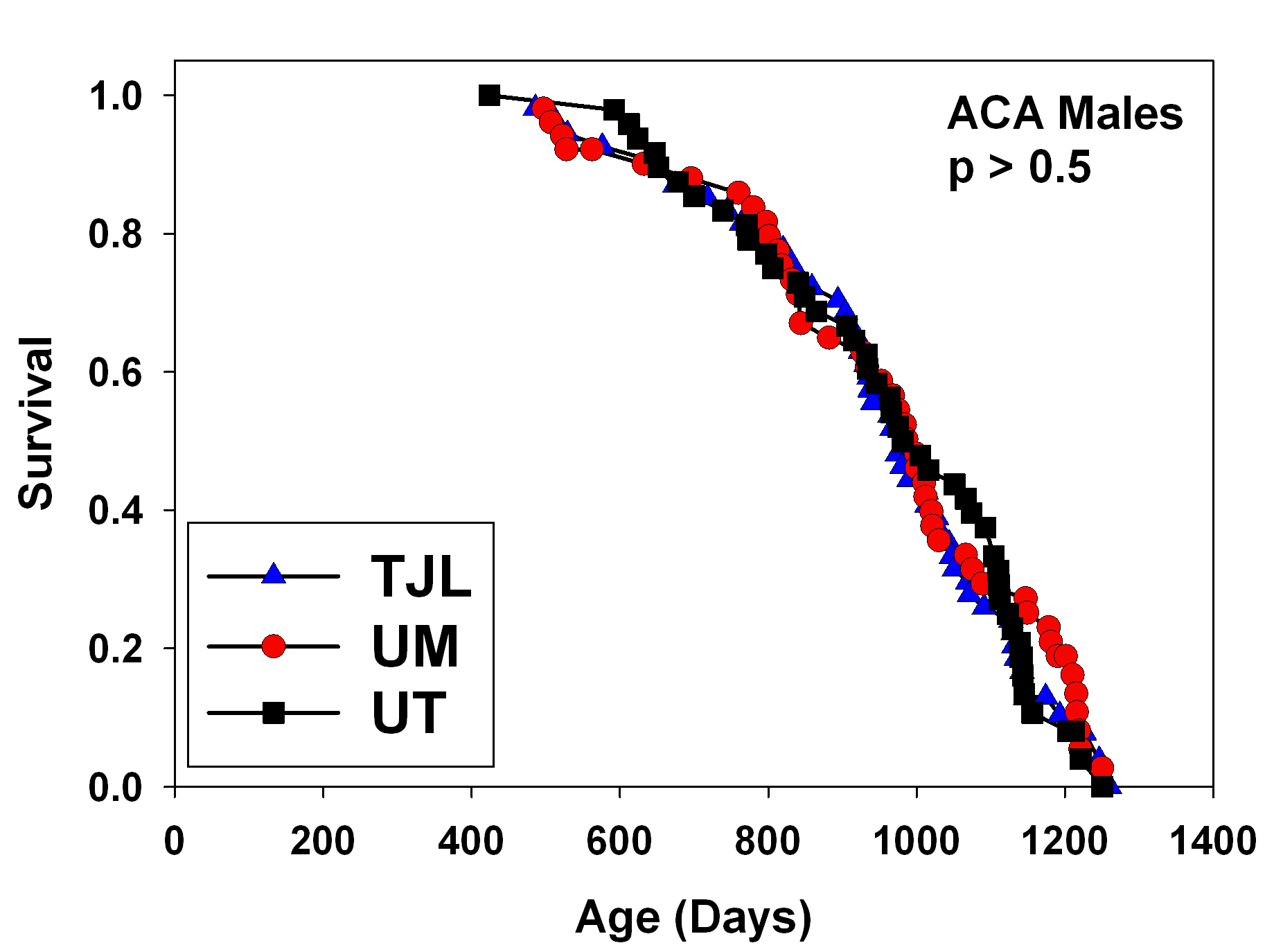 | 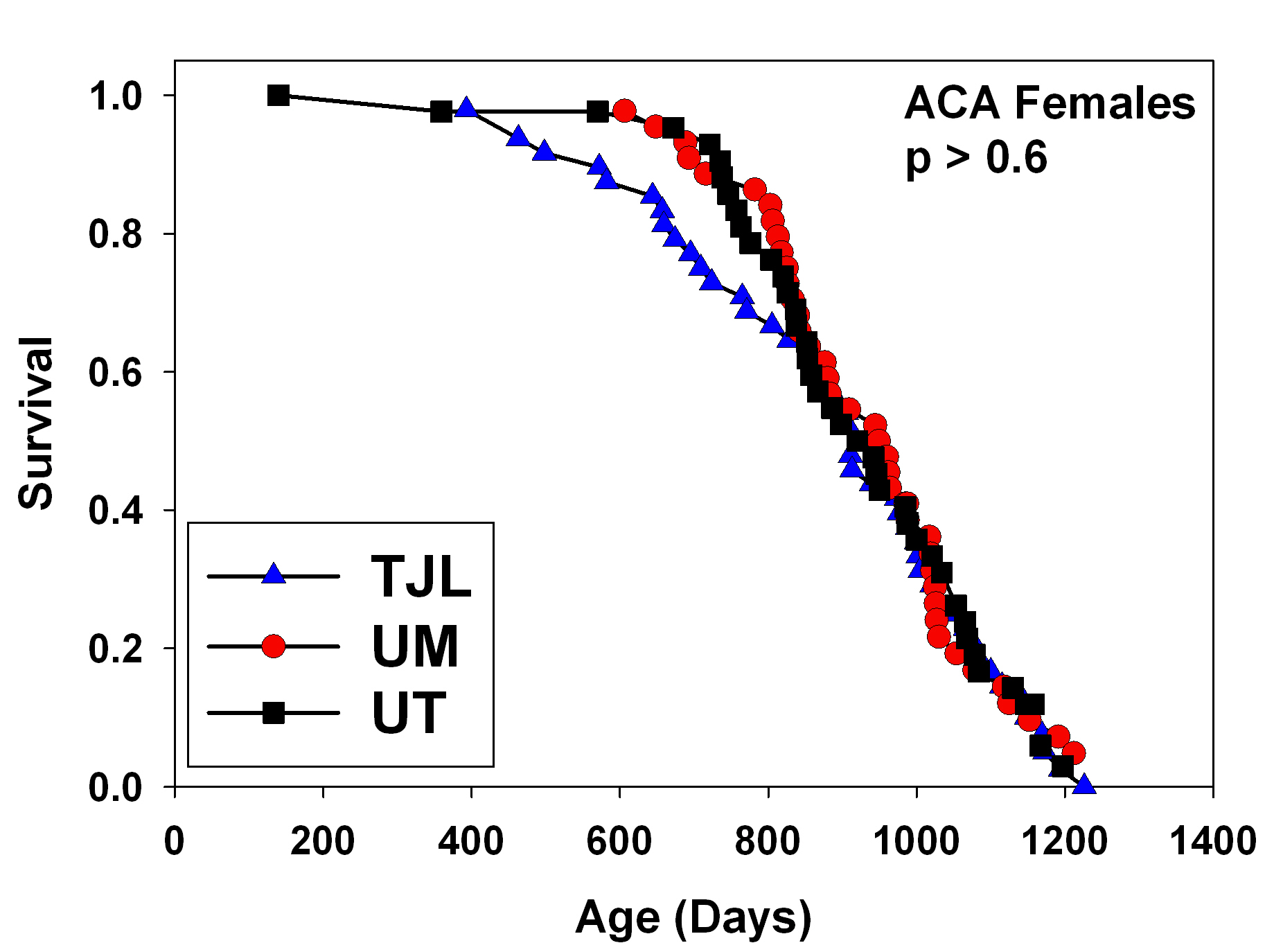 |

**Fig S2** ACA, EST and MB treatment: weights for UM-HET3 controls and males (A–C) and females (D–F) at TJL (A,D), UM (B,E) and UT (C,F).

| **(A)** | **(B)** | **(C)** |
| --- | --- | --- |
| 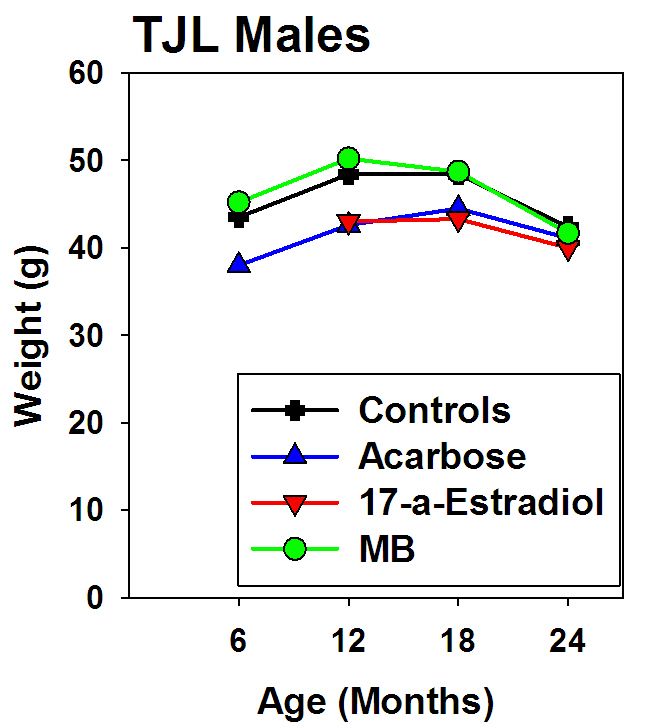 | 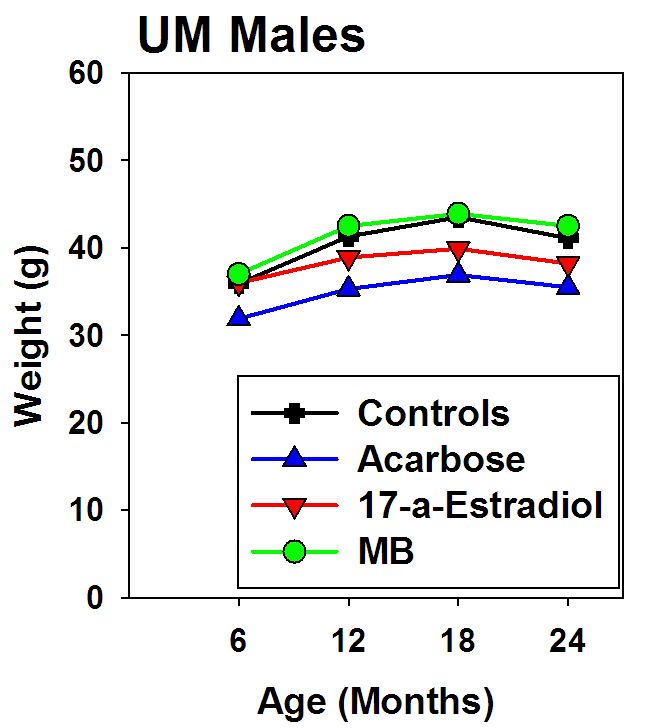 | 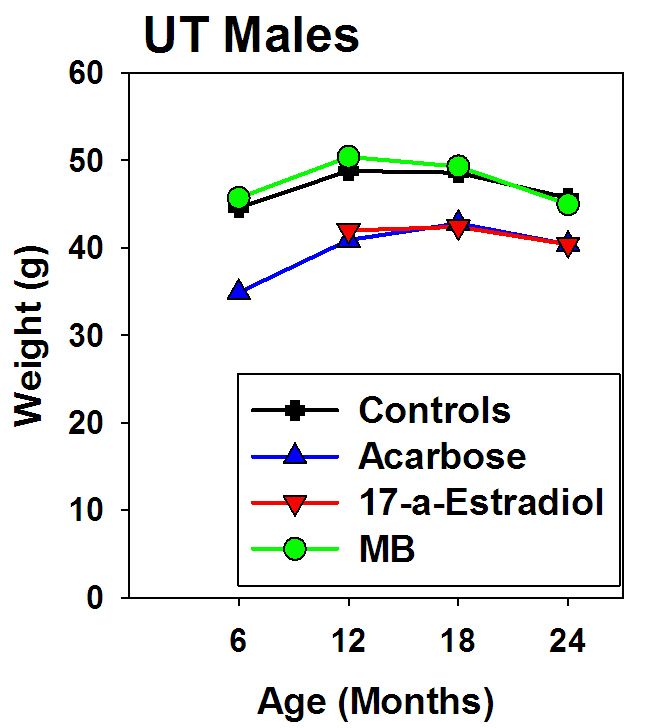 |
| **(D)** | **(E)** | **(F)** |
| 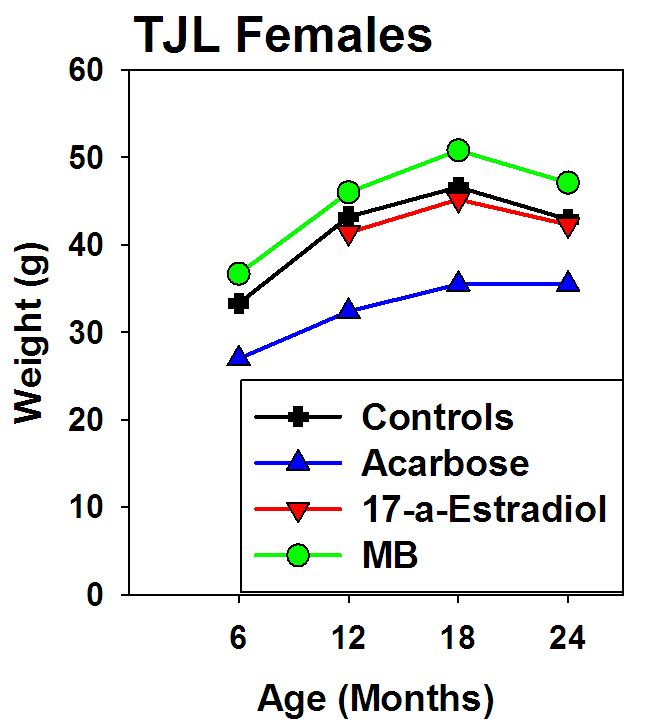 | 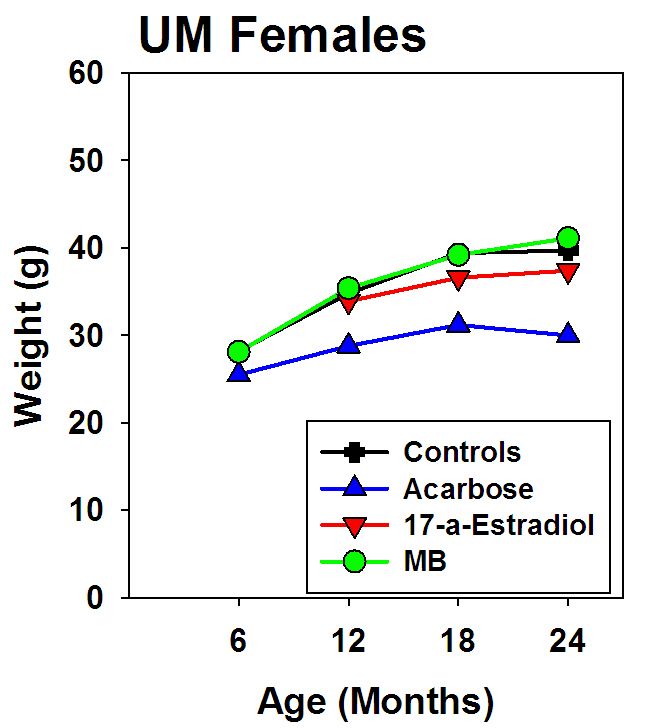 | 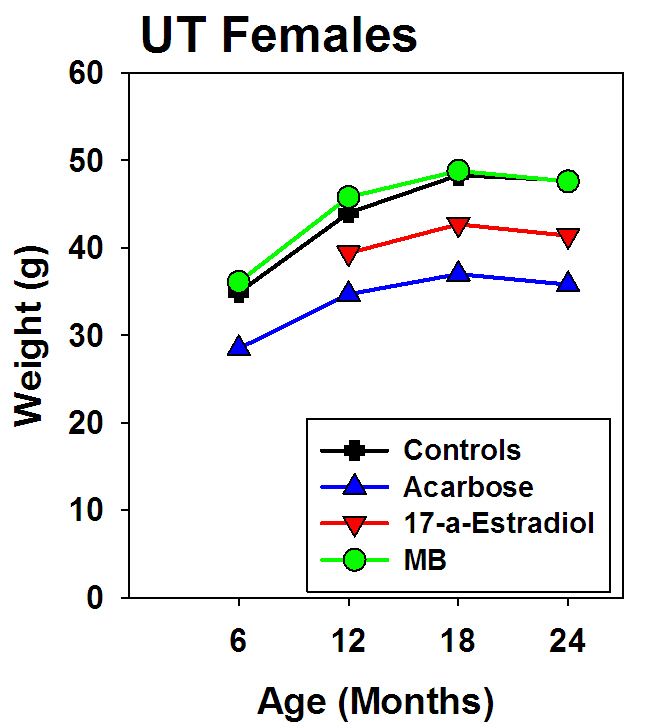 |

**Fig S3** EST treatment: Survival for UM-HET3 control females pooled across sites (A) and EST-treated females at TJL (B), UM (C), and UT (D).

| **(A)** | **(B)** |
| --- | --- |
| 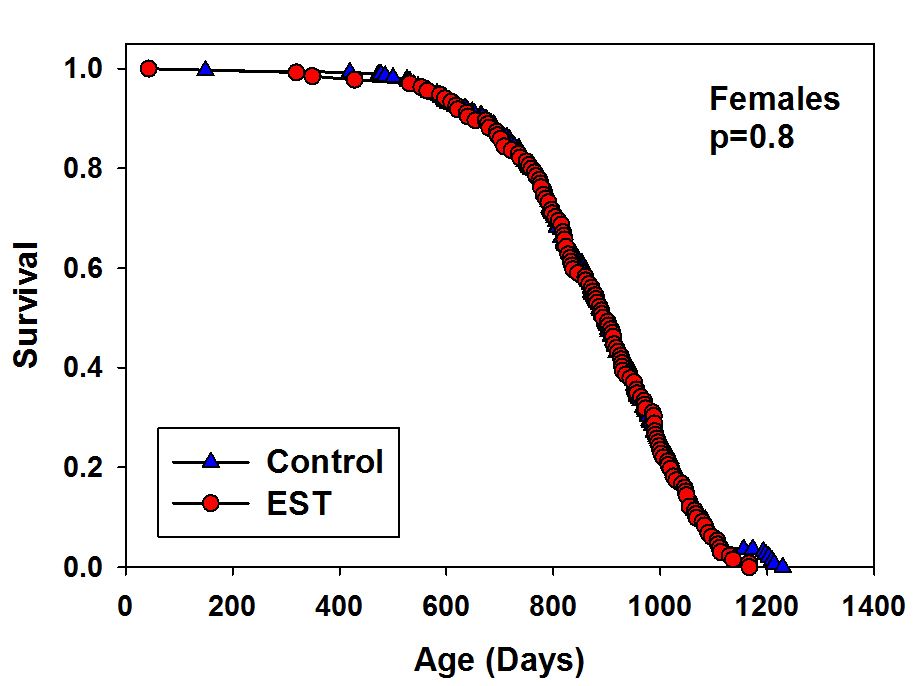 | 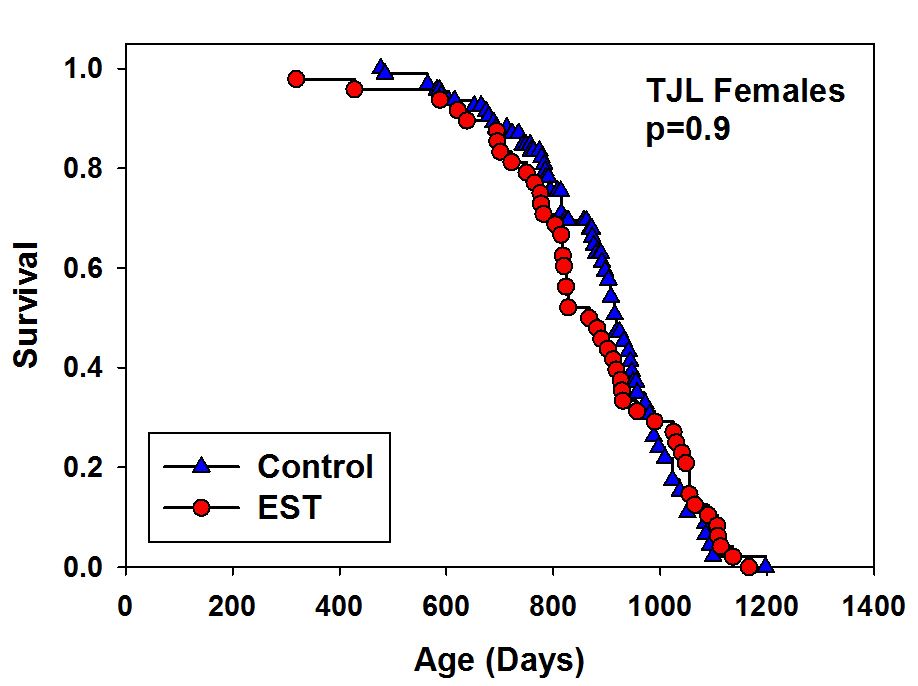 |
| **(C)** | **(D)** |
| 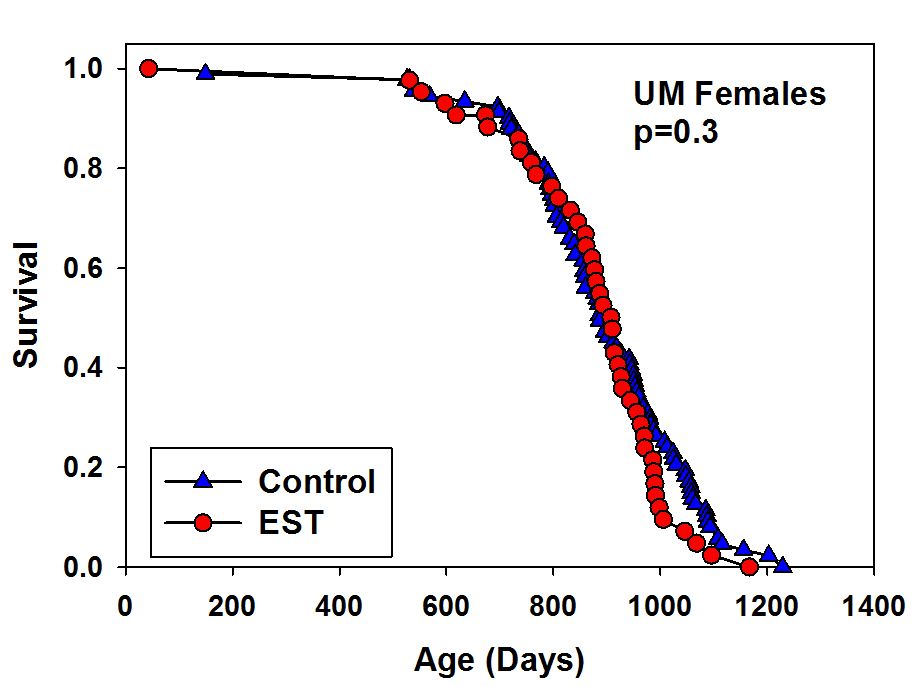 | 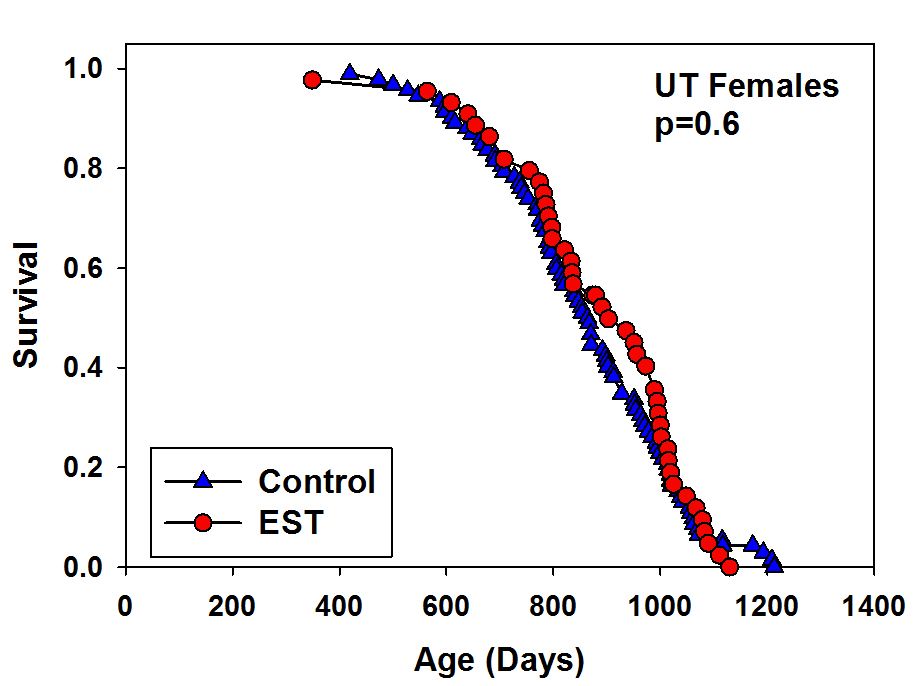 |

**Fig S4** NDGA treatment: Interim survival for UM-HET3 controls and NDGA-treated males at TJL (A), UM (B), and UT (C), and UM-HET3 controls and NDGA-treated females at TJL (D), UM (E), and UT (F).

| **(A)** | **(B)** | **(C)** |
| --- | --- | --- |
| 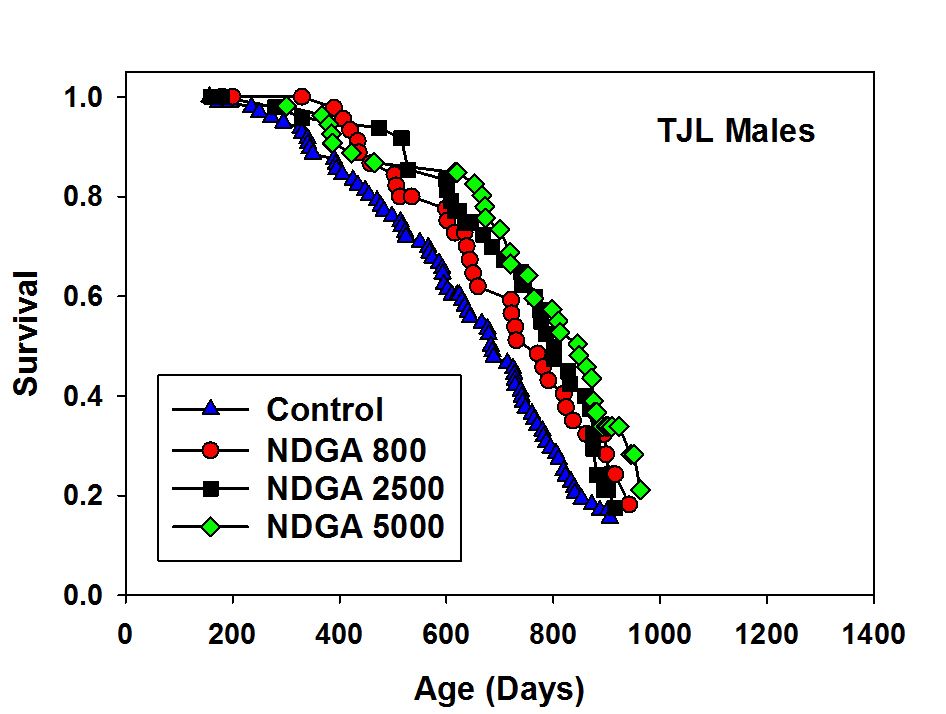 | 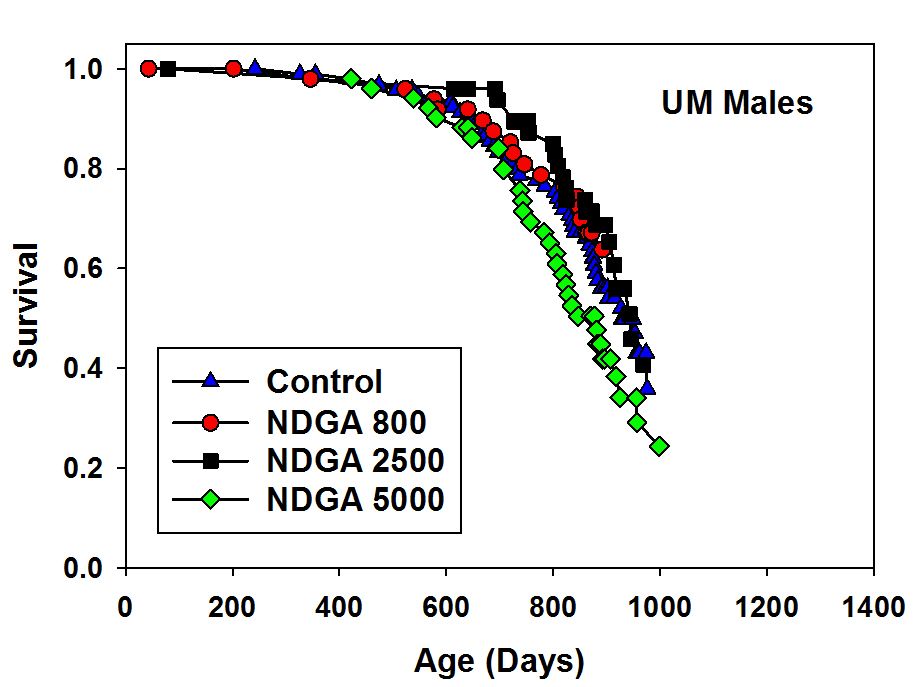 | 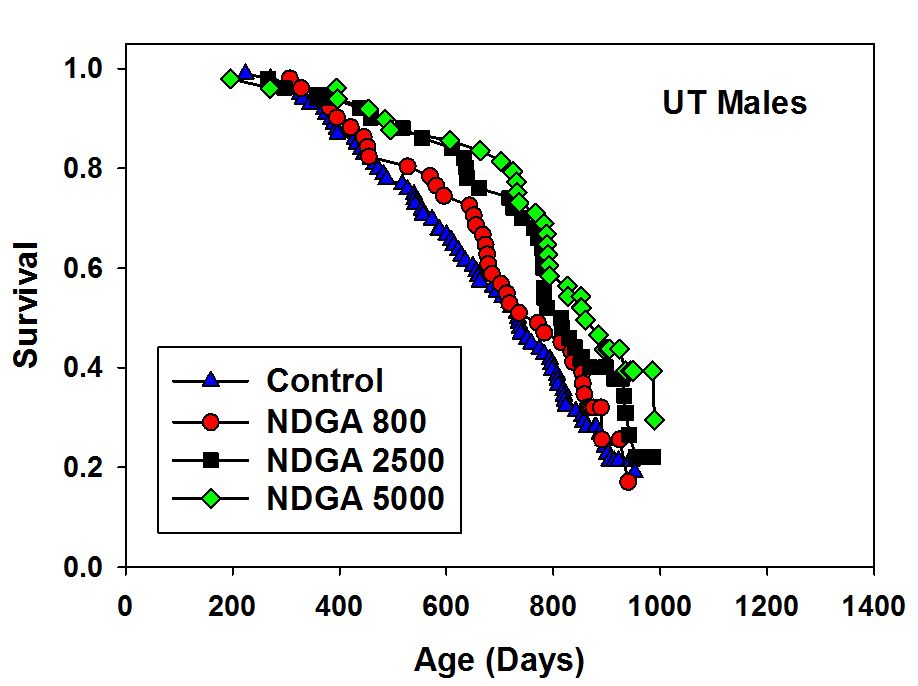 |
| **(D)** | **(E)** | **(F)** |
| 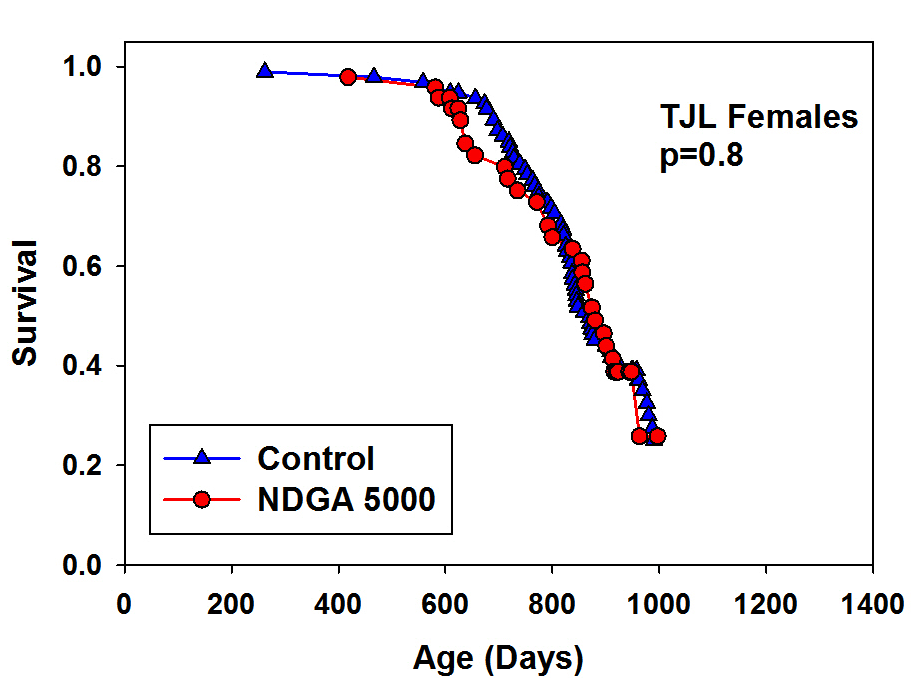 | 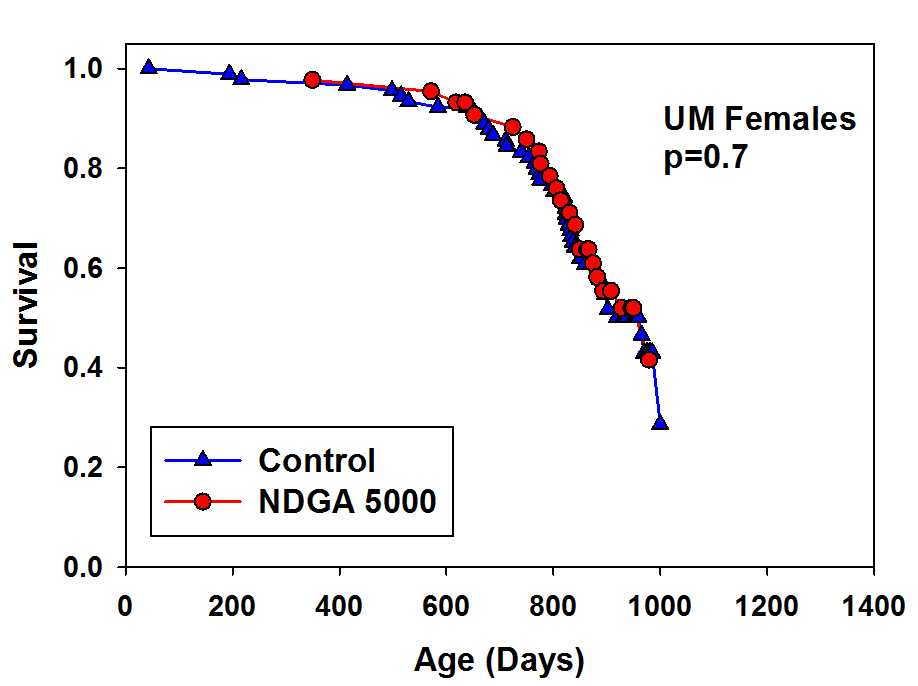 | 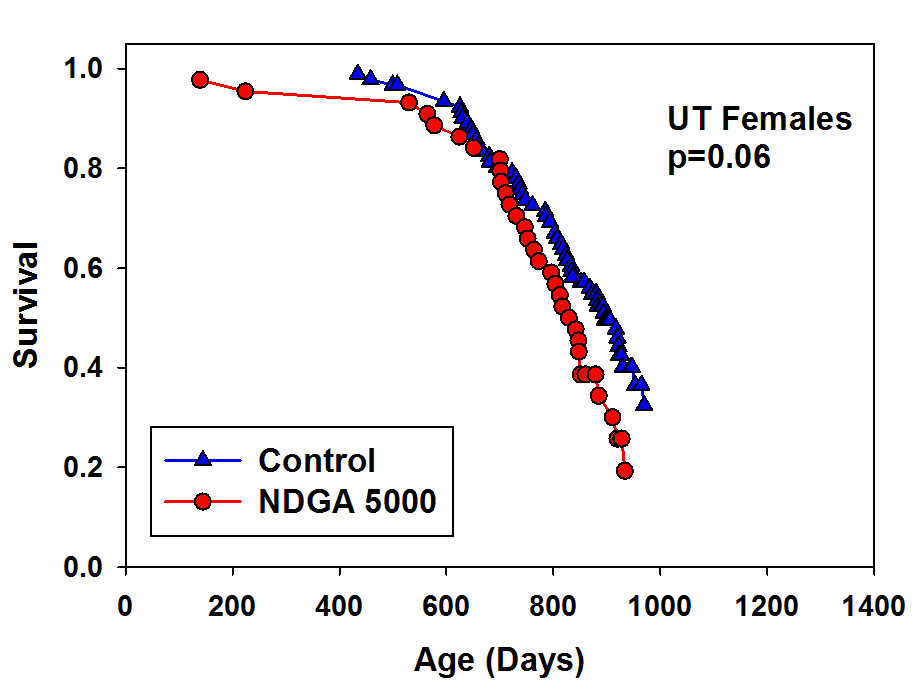 |

**Fig S5** NDGA levels in plasma of UM-HET3 controls and males treated with high (5000 ppm), middle (2500 ppm), or low dose (9800 ppm) of NDGA in the diet (A), and UM-HET3 controls and females treated with high dose of NDGA (B).

| **(A)** | **(B)** |
| --- | --- |
|  |  |

**Fig. S6** NDGA treatment: Body weights for UM-HET3 controls and males treated with high, middle, and low dose of NDGA (A), and for UM-HET3 controls and females treated with high dose of NDGA (B).

| **(A)** | **(B)** |
| --- | --- |
| 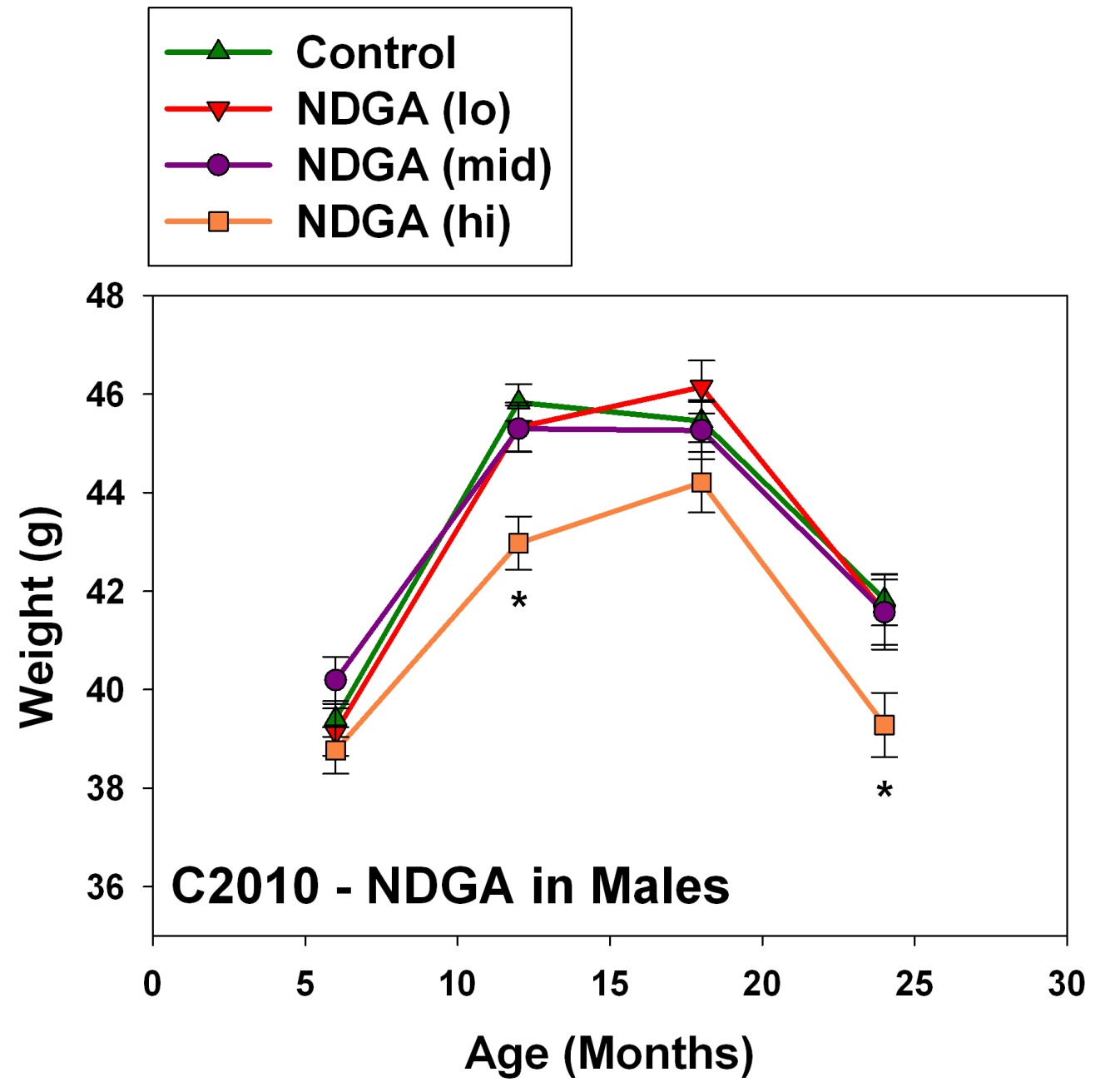 | 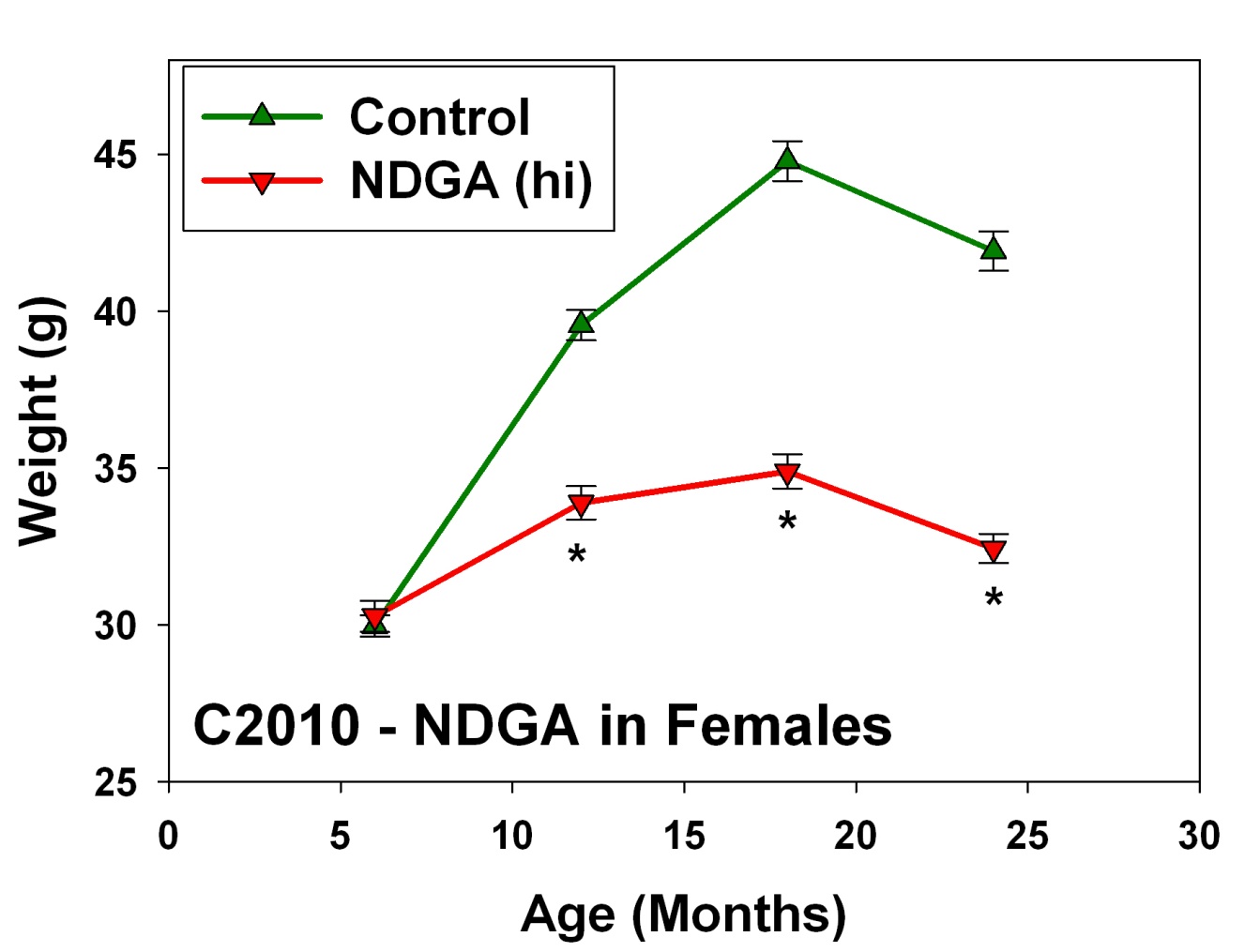 |

**Fig S7** MB treatment: Survival curves for UM-HET3 controls and mice fed MB in the diet for males at TJL (A), UM (B), and UT (C), and for females at TJL (D), UM (E), and UT (F).

| **(A)** | **(B)** | **(C)** |
| --- | --- | --- |
| 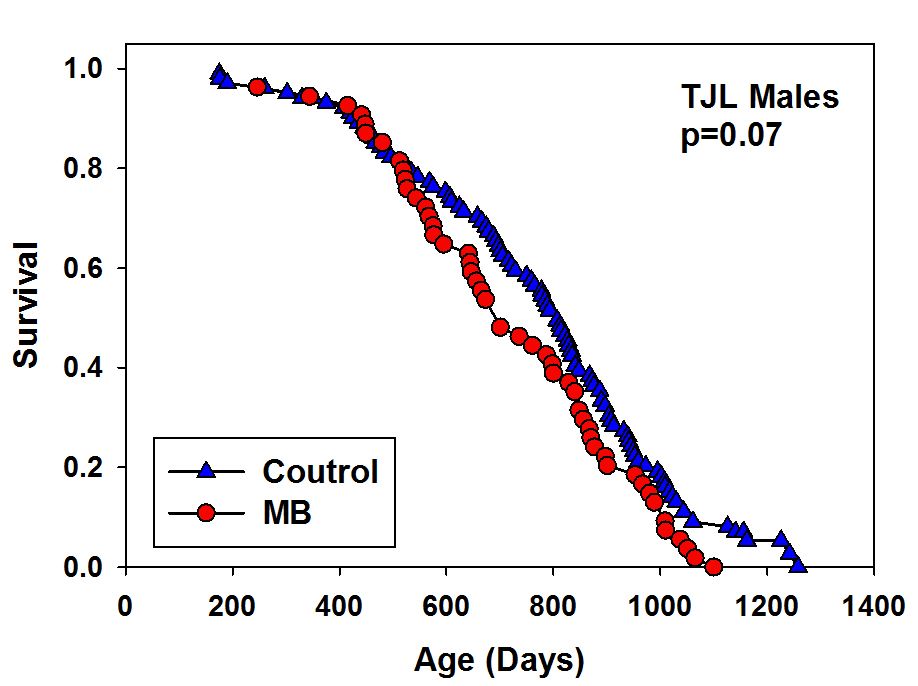 | 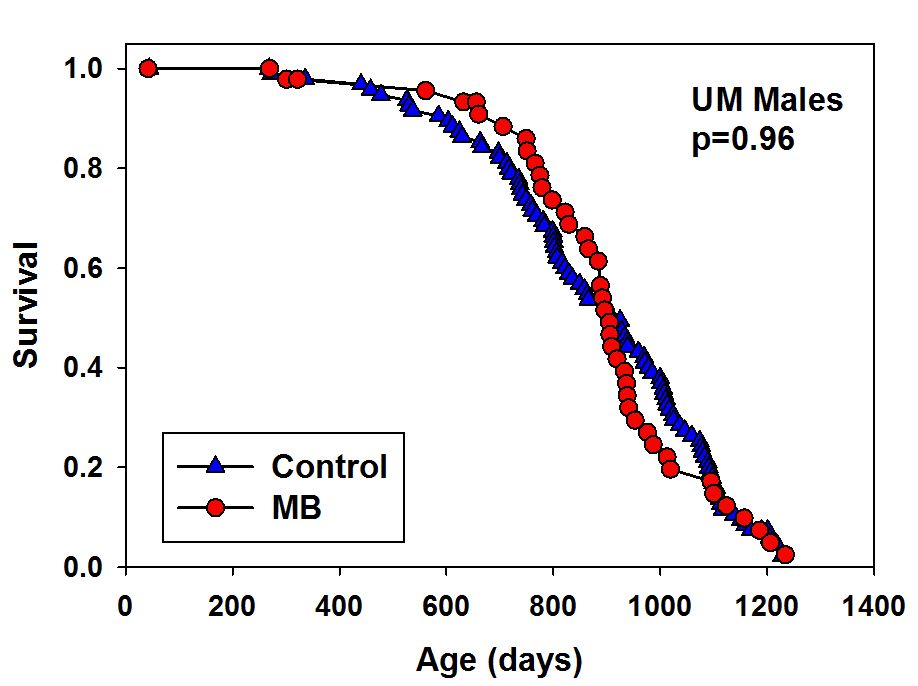 | 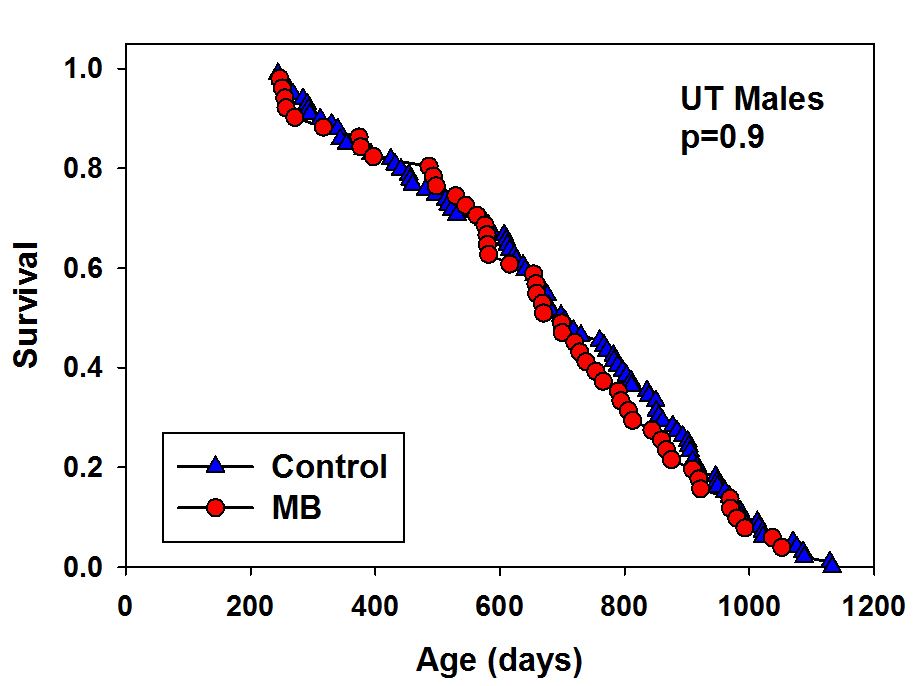 |
| **(D)** | **(E)** | **(F)** |
| 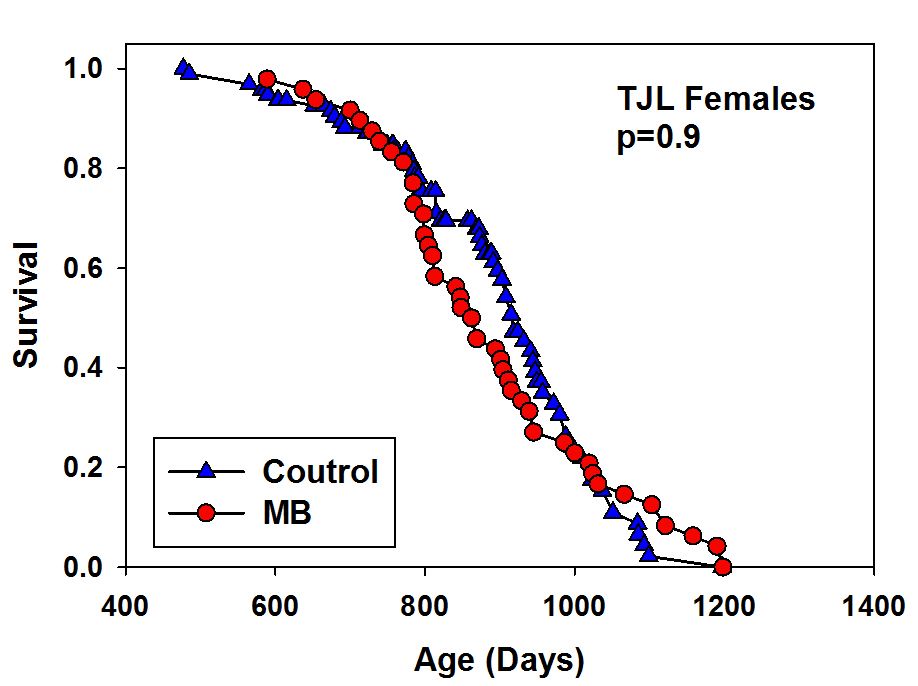 | 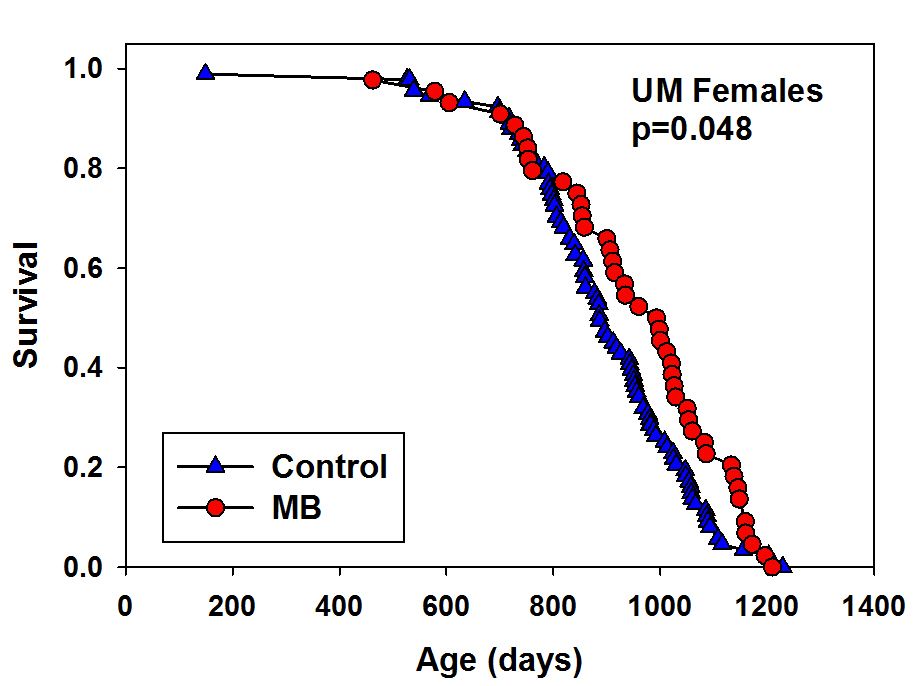 | 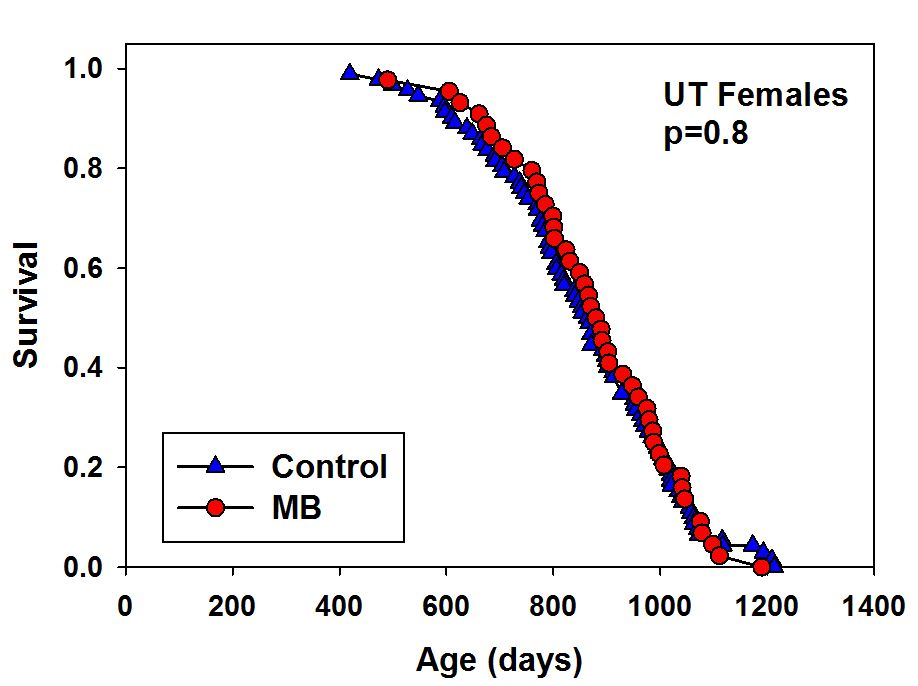 |

**Table S1** ACA and EST treatment: Inferred causes of death in aging UM-HET3 controls and ACA- and EST-treated males and females

| Type of lesion | Controls | ACA-treated mice | EST-treated mice | Total lesions |
| --- | --- | --- | --- | --- |
| Amyloidosis |  |  | 1 | 1 |
| Atrial thrombosis | 2 |  |  | 2 |
| Autolysis | 4 | 2 | 2 | 8 |
| Carcinoma |  | 1 |  | 1 |
| Fibrosarcoma |  | 3 | 1 | 4 |
| Granulomatous hepatitis |  | 1 |  | 1 |
| Hemangiosarcoma | 7 | 7 | 3 | 17 |
| Hemorrhage |  |  | 1 | 1 |
| Hepatocellular carcinoma | 3 | 2 | 2 | 7 |
| Hydrometra |  | 1 | 1 | 2 |
| Hypertension | 1 |  |  | 1 |
| Lung adenocarcinoma | 7 | 10 | 7 | 24 |
| Lymphoma | 13 | 14 | 23 | 50 |
| Mammary adenocarcinoma |  | 4 | 2 | 6 |
| Mammary tumor | 1 |  |  | 1 |
| Metastatic adenocarcinoma | 1 |  |  | 1 |
| Myocardial (ventricle) thrombosis | 1 |  |  | 1 |
| Nephritis | 1 |  |  | 1 |
| Open | 8 | 4 | 5 | 17 |
| Prostate adenocarcinoma |  |  | 1 | 1 |
| Renal amyloidosis |  | 1 | 1 | 2 |
| Sarcoma NOS |  | 1 |  | 1 |
| Septicemia |  |  | 1 | 1 |
| Steatitis |  | 1 | 1 | 2 |
| **Grand Total** | **49** | **52** | **52** | **153** |
| Diagnoses | 37 | 46 | 45 | 128 |

Of the 153 cases analyzed, 8 had severe autolysis, 17 were left “open,” with no evidence for a single major cause of death, and 128 were given diagnoses. Of the 153 cases, distribution among sites was not equal: UM = 60, UT = 67 TJL = 26 (many of the TJL mice were too severely autolyzed to allow analysis). Table was prepared by J. Erby Wilkinson.

**Table S2** Survival in UM-HET3 control and male mice that were treated with ACA, EST and MB in the diet and that lived at least 600 days

|  | Males with lifespans >600 days of age | | | | All males | | |
| --- | --- | --- | --- | --- | --- | --- | --- |
|  | Median lifespan (d) | Median Increase (%) | Log-rank p-value | *n* | Median lifespan (d) | Median increase (%) | Log-rank p-value |
| TJL |  |  |  |  |  |  |  |
| Controls | 873 |  |  | 76 |  |  |  |
| ACA | 980 | 12 | 0.007 | 50 |  |  |  |
| EST | 941 | 8 | 0.22 | 37 |  |  |  |
| MB | 848 | -3 | 0.17 | 35 |  |  |  |
| UM |  |  |  |  |  |  |  |
| Controls | 938 |  |  | 86 |  |  |  |
| ACA | 1000 | 7 | 0.055 | 44 |  |  |  |
| EST | 951 | 1 | 0.28 | 43 |  |  |  |
| MB | 906 | -3 | 0.72 | 42 |  |  |  |
| UT |  |  |  |  |  |  |  |
| Controls | 850 |  |  | 67 |  |  |  |
| ACA | 1005 | 18 | < 0.0001 | 47 |  |  |  |
| Est | 911 | 7 | 0.04 | 44 |  |  |  |
| MB | 806 | -5 | 0.8 | 32 |  |  |  |
| Pool |  |  |  |  |  |  |  |
| Controls | 887 |  |  | 229 | 807 |  |  |
| ACA | 1000 | 13 | 0.0001 | 141 | 984 | 22 | < 0.0001 |
| EST | 934 | 5 | 0.011 | 124 | 900 | 12 | 0.002 |
| MB | 870 | -2 | 0.42 | 109 | 790 | -2 | 0.27 |
| Mean |  |  |  |  |  |  |  |
| Controls | 887 |  |  |  |  |  |  |
| ACA | 995 | 12 |  |  |  |  |  |
| EST | 934 | 5 |  |  |  |  |  |
| MB | 853 | -3 |  |  |  |  |  |

Male survival statistics were recalculated after eliminating all deaths at ages earlier than 600 days. This has little effect on the values for the cohorts, which were presented in Table 1. “Pool” describes data from all three sites pooled for statistics. “Mean” gives the average of the mean values from each site.
